# The Psychology of EdTech Nudging: Persuasion, Cognitive Load, and Intrinsic Motivation

**DOI:** 10.3390/ejihpe15090179

**Published:** 2025-09-06

**Authors:** Stefanos Balaskas, Ioanna Yfantidou, Theofanis Nikolopoulos, Kyriakos Komis

**Affiliations:** 1 Department of Physics, School of Sciences, Democritus University of Thrace, Kavala Campus, 65404 Kavala, Greece; 2 Department of Business and Management, Liverpool John Moores University (LJMU), Rodney Street, Liverpool L3 5UL, UK; i.yfantidou@ljmu.ac.uk; 3 School of Social Sciences, Hellenic Open University, 18 Parodos Aristotelous St., 26335 Patras, Greece; nikolopoulos.theofanis@ac.eap.gr; 4 Department of Electrical and Computer Engineering, School of Engineering, University of Patras, 26504 Patras, Greece; up1079081@upatras.gr

**Keywords:** persuasive design, digital nudging, personalization, intrinsic motivation, cognitive overload, perceived autonomy, educational technology, structural equation modeling

## Abstract

With increasing digitalization of learning environments, concerns regarding the psychological effect of seductive interface design on the motivational level and cognitive health of learners have been raised. This research investigates the effects of certain persuasive and adaptive design elements, i.e., Perceived Persuasiveness of Platform Design (PPS), Frequency of Nudge Exposure (NE), and Perceived Personalization (PP), on intrinsic motivation in virtual learning environments (INTR). We draw on Self-Determination Theory, Cognitive Load Theory, and Persuasive Systems Design to develop and test a conceptual model featuring cognitive overload (COG) and perceived autonomy (PAUTO) as mediating variables. We used a cross-sectional survey of university students (*N* = 740) and used Partial Least Squares Structural Equation Modeling (PLS-SEM) for data analysis. The findings show that all three predictors have significant impacts on intrinsic motivation, with PP as the strongest direct predictor. Mediation analyses produced complementary effects for NE and PP in that these traits not only boosted motivation directly, but also autonomy, and they decreased cognitive overload. Alternatively, PPS showed competitive mediation, boosting motivation directly but lowering it indirectly by increasing overload and decreasing autonomy. Multi-Group Analysis also revealed that such effects differ by gender, age, education, digital literacy, exposure to persuasive features, and use frequency of the platform. The results underscore the imperative for educational technology design to reduce cognitive load and support user control, especially for subgroups at risk. Interface designers, teachers, and policymakers who are interested in supporting healthy and ethical digital learning environments are provided with implications. This work is part of the new generation of research in the field of the ethical design of impactful education technologies, focusing on the balance between motivational-enabling functions and the psychological needs of users.

## 1. Introduction

Internet environments are increasingly being crafted to nudge user behavior through subtle data-based changes that do not claim to dictate decision but subtly influence them (De Ridder et al., 2022; Kalvit & Singhvi, 2025; Wachner et al., 2020). Across domains such as public health, personal finance, and education, nudges aim to guide users toward beneficial choices while preserving autonomy (Brown et al., 2023; Chapman et al., 2025). However, the recent surge of nudging into e-learning, more specifically, into persuasive learning technologies, has triggered critical discussions on maintaining the equilibrium between stimulating engagement and using psychological pressure. While there is as much evidence for nudges increasing learning engagement in learning contexts (Brown et al., 2023; Inocencio, 2018), there are still concerns about ethical acceptability, transparency, and learners’ autonomy when faced with intrusive or persistent persuasive interventions (Ning et al., 2025; Shi et al., 2025).

The increased implementation of persuasive educational systems (PESs), a merger of learning analytics, gamification, and behavioral nudges, highlights the move towards engaging with students as learners and digital consumers (Wenker, 2022; Yin et al., 2021). For instance, gamified micro-learning systems and chatbot-assisted learning platforms have been shown to have positive influences on intrinsic motivation through focus on perceived value and choice, whereas nudging interventions have been able to effectively enhance online engagement and task completion (Brown et al., 2023; Yin et al., 2021). Yet, the efficacy of these types of systems has not been consistent. As more recent studies of tourism and sustainability suggest, the rate and type of nudges must be well-tuned not to cause user fatigue or backfiring (Zhang, 2023). Likewise, in educational settings, a skewed mix of gamification features, routine reminders, or social comparison items may debase learner autonomy, eliciting resistance or disaffection instead of long-term motivation (Jin & Bridges, 2014; Murillo-Muñoz et al., 2021).

The second fundamental challenge is the sense of autonomy and psychological cost of persuasion. Wachner et al., 2020, show that default nudges are viewed as threats to autonomy when salient, but social norm nudges will be less likely to provoke adverse responses. This difference in perception is vital in education, where autonomy is a fundamental impeller of intrinsic motivation and self-regulated learning (Murillo-Muñoz et al., 2021). Only recently has the issue highlighted that, while engagement can be encouraged through persuasive means, its design must find a balance between learners’ psychological states, cognitive load, and motivation (Sheetal & Singh, 2023; Srivastava et al., 2024). Without such a balance, nudging risks obscuring the difference between “smart persuasion” and digital pressure, undermining both student welfare and the validity of the learning environment.

Personalization and adaptive nudging research identifies promising directions but is underdeveloped in the context of education. While marketing and consumer science have been successful with social norm messages and personalized nudges in altering behavior (Shi et al., 2025; Smith et al., 2019), educational technology has frequently been lacking in implementing subtlety in responding to the needs, preferences, or autonomy orientation of students. Even with efforts at personalization, it sometimes does not pay, as indicated by Plak et al., 2023, who discovered that personalizing nudges to students’ levels of motivation did not necessarily translate to greater participation in online tests. The gap needs more advanced theory-based models that also fuse behavioral science, motivation theory, and persuasive technology design.

The present research fills these gaps by investigating the invisible marketing logic behind EdTech nudges, and how the visual designs of streaks, game-like badges, reminders of progress, and personalized notifications can function not only as learning tools, but also as influence methods able to shape students’ mental experience. While the existing literature has explored nudging in learning as an engagement strategy, it is limited in the assessment of how it will induce psychological pressure, cognitive overload, or opposition to learner autonomy (Ning et al., 2025; Shi et al., 2025). In its simulation of the interaction between perceived persuasiveness, psychological pressure, and intrinsic motivation, the paper describes a systematic examination of when and how persuasive EdTech design promotes or impedes quality learning experiences. We employ “nudging” in this article to describe light-touch choice architecture that guides behavior by framing options in a manner that impacts behavior without closing off alternatives or altering incentives in any significant way (cf. behavioral economics). “EdTech nudges” in edutech thus describe platform-embedded data-driven cues such as reminders, streak counters, badges, default options, and customized recommendations that promote study continuity or goal advancement while being sensitive to freedom of user choice. Throughout, we contrast nudges with coercive mandates and stand-alone notices by their deliberate purpose to shape choices under retained autonomy.

Through its approach, this study also adds to the debate regarding the ethics, transparency, and effectiveness of behavioral interventions to support online learning (Jin & Bridges, 2014; Murillo-Muñoz et al., 2021). It combines cross-disciplinary research in behavioral nudging, persuasive systems, and Self-Determination Theory to create a conceptual framework, closing the gap between quantitative measures of engagement (e.g., logins, streaks, completion rates) and qualitative aspects of learning motivation and autonomy. By considering these mechanisms of the mind, this paper presents a more critical consideration of how design principles for persuasion motivated by market rationality themselves must be redesigned with care for educational use so as not to undermine the very outcomes that they aim to maximize.

To foreshadow the main results, the findings of this study validated that Perceived Personalization, persuasive platform design, and Nudge Exposure frequency each positively influence students’ intrinsic motivation in online learning contexts to a significant extent. Mediation analyses also showed that personalization and nudging have both direct and indirect positive influences through autonomy enhancement and cognitive overload reduction, while persuasive design, though motivation, also created psychological pressure, through which competitive mediation effects occurred. Multi-group comparisons indicated that these dynamics differ across demographic and contextual dimensions of gender, age, digital literacy, and exposure to persuasive features. The results provide a detailed explanation of how digital design impacts student motivation and guides the development of more ethically conscious and psychologically supportive educational technologies.

The rest of this paper is arranged as follows: Section 2 presents an overview of the theory and empirical studies on persuasive design, personalization, and motivational dynamics in online learning environments. Section 3 states the conceptual framework and research hypotheses of this study. Section 4 defines the methodology, participant recruitment, instruments for measurement, and statistical processes applied to SEM and Multi-Group Analysis. Section 5 lists the key findings, including direct effects, mediation processes, and subgroup differences. Section 6 addresses practical implications for educators, designers, and policymakers seeking to maximize digital learning platforms. Section 7 concludes with a final overview of key findings, study limitations, and directions for future research.

## 2. Literature Review

### 2.1. Persuasive Design and EdTech Nudges

Persuasive design has become mainstream in digital learning environment design, especially with educational technologies continuing to embrace strategies and tactics from marketing and behavior change systems (Akgün & Topal, 2018; Alslaity et al., 2023; Huang et al., 2024; Knight, 2025). Rooted in the assumptions of Persuasive Systems Design (PSD) principles, gamification systems, and behavioral psychology, EdTech platforms now incorporate features like badges, streak counters, pop-up reminders, and social comparison functionalities to shape learner engagement and motivation. Yet, this ubiquity of persuasive features necessarily invites questions regarding their psychological impacts, particularly in learning environments where long-term autonomy and cognitive well-being are most essential.

Increasing numbers of studies have attempted to trace the impact of persuasion strategies on student motivation, frequently with respect to Self-Determination Theory (SDT). (F. A. Orji et al., 2024), illustrating that strategies such as self-monitoring and commitment-consistency are more aligned with SDT’s competence and autonomy motivations than more controlling external aspects such as competition or social comparison. These results suggest that not all persuasive design methods are equally effective or healthy in a psychological sense; instead, the extent to which a design promotes intrinsic motivation is moderated by the motivational needs congruence of the users.

This is encapsulated in comprehensive research on user types that creates notable differences in people’s susceptibility to persuasive tactics. For example, (R. Orji et al., 2018) found that ‘players’ and ‘socialisers’—on the basis of the HEXAD gamification model of user types—are highly responsive to reward-based and social approaches, while ‘disruptors’ dislike externally forced structures like goal-setting and punishment. Such distinctions render personalization vital in persuasive learning systems. (Alslaity et al., 2023), in a systematic review of personalization-based persuasive technologies, observe that, while gamification and PSD principles are used extensively in education, their impact is context-dependent and usually moderated by user characteristics like personality, initial motivation, and nudging susceptibility.

Individualized EdTech systems are increasingly popular but their cognitive and ethical effects from the use of persuasive strategies are still in their infancy. (Murillo-Muñoz et al., 2021) observe that persuasive learning systems are used without reflection on how persuasion converges with learning goals, cognitive load, or user empowerment. Parallel to this, (Huang et al., 2024) present empirical support that perceived persuasiveness acts as a mediator of the gamified reward’s influence on sustainable mobile app users’ behavior, indicating that persuasion is not merely a superficial design aspect, but an internalized psychological process capable of enabling as well as limiting action.

From a critical design perspective, the use of persuasive techniques in educational settings needs to be treated responsibly. (Martin & Kwaku, 2019) state that reciprocity, consistency, and liking are generally persuasive but are highly context-dependent in terms of acceptability and efficacy across HEXAD profiles. Additionally, (Sheetal & Singh, 2023) point towards severe ethical issues by observing that, in gamified retail environments, persuasive strategies may tip towards manipulation, reducing trust and control, comparisons that might be pertinent in EdTech scenarios where consent and knowledge of students are typically superficial.

The case for ethically grounded adaptive persuasive systems is supported by emerging machine learning-driven personalization trends. (Knutas et al., 2019) promote rule-based systems mapping persuasive content to user profiles without inducing psychological misalignment. This supports the (McCarthy et al., 2024) report, which foregrounds socio-cognitive representations and user journeys over static demographic classifications. These methods redirect the discussion from general persuasion to context-aware nudging, not only what captures users, but also what maintains cognitive autonomy and learning efficiency.

Even with these encouraging advances, the education field falls behind the likes of health and sustainability in systematically establishing the impact of persuasion interventions. Most education research, (Murillo-Muñoz et al., 2021) observe, uses a variety of persuasion strategies without assessing their individual or overall impact. In addition, while immersive learning spaces (Wiafe et al., 2024) and mobile learning spaces (Za et al., 2022) have identified more results pertaining to persuasive features, these are less likely to sever the mechanisms of psychological pressure or the effect of Perceived Personalization, which is highly important in explaining unintended effects like digital fatigue or crowding-out of motivation.

Recent studies also identify large language model (LLM)-powered chatbots as credible and responsive agents in learning contexts. LLM-powered classroom flipping and peer-questioning assistance, for instance, can act as just-in-time nudgers, making visible cues, prompts, and follow-up questions that nudge participation at scale (Wiafe et al., 2024). While such agents can enable engagement, they amplify ongoing debates concerning transparency, manipulation, and learner control in digital nudging (Ng et al., 2024). In SDT, the same support feels autonomy-supportive (choice, rationale, reversibility) or controlling (invisible intent, overprompting). In agreement with CLT, poorly calibrated chatbot prompts risk extraneous cognitive load. These tensions require not only measuring persuasive chatbots on engagement metrics, but also on autonomy and cognitive imprints (Wiafe et al., 2024). In our model, LLM chatbots utilize Perceived Persuasiveness of Platform Design (PPS) via anthropomorphic authority cues and framing appeals; Nudge Exposure (NE) via prompt rhythm, rate, and initiative; and provide Perceived Personalization (PP) via context-sensitive explanation and task adaptation. Therefore, chatbot design decisions are expected to undergo cognitive overload (COG) and perceived autonomy (PAUTO) to influence intrinsic motivation (INTR), explaining why dialogic agents can facilitate as well as discourage motivation. Locating this technology within ongoing controversies also implies transparency by design, issuing “why this/why now?” rationales of prompts, openly visible nudge settings, snooze/opt-out under user management, and graduated proactivity in order to keep persuasion autonomy-supportive and cognitively unobtrusive. Methodologically, a signal of cost and value both require mixed measures across engagement: (i) quantitative measures (valenced perceived persuasiveness scales separating beneficial from controlling influence; prompt cadence and interruption logs; dismissal/override rates; mean cognitive-load and autonomy measures) and (ii) qualitative measures (think-alouds, trace-based interviews) to access reports of control and manipulation. Lastly, considering the subgroup-sensitive effects of persuasion, LLM nudging should be compared using multi-group contrasts (e.g., digital literacy, experience with persuasive features, gender/age), just as our MGA approach, to allow for proactiveness and personalization intensity to be tuned to learner profiles and project dose–response “rate limits” to be determined to prevent overloading without undermining autonomy. Supporting this argument, a 3-week comparison study with secondary school students (*N* = 74) concluded that a generative AI self-regulated learning (SRLbot) chatbot bested a rule-based one in science knowledge, behavioral engagement, and motivation; chatbot interaction numbers significantly predicted gains in SRL; and students credited personalized support and flexibility for benefits (Ng et al., 2024). Students also indicated less learning anxiety and more consistent study habits with the generative AI design, which implies that transparent user-controllable LLM prompts can act as autonomy-supportive nudges when timely and sensitive in their use of context, with a note of warning that cadence and cognitive load should always be monitored so as not to control or overwhelm.

This research attempts to fill these gaps by empirically investigating the two psychological mechanisms elicited by persuasive design in intelligent learning spaces, i.e., perceived psychological pressure and autonomy, and how they influence intrinsic motivation. In the process, it helps expand knowledge about how persuasive strategies not only engage more, but influence learners’ emotional and mental conditions for better or worse. It also underscores the importance of ethical-by-design principles in the learning UX, reaffirming the excellence of coupling persuasive factors with the users’ personality and motivational theories, as opposed to boilerplate gamification templates. Building on the preceding review of persuasive design in EdTech, we next consider how psychological pressure and cognitive load shape users’ responses to such features.

### 2.2. Psychological Pressure and Cognitive Load

The sudden shift to online education amid the COVID-19 pandemic heightened psychological tension and cognitive load for every stakeholder in education, which revealed inherent limits in virtual learning environments (Antoniadou et al., 2022; Appel & Fernández, 2022; Bozkurt et al., 2020; Cheng et al., 2023). Exponential scholarship conveys how emergency remote teaching (ERT) resulting from the pandemic incited psychological loads on both students and employees, not just derailing pedagogical continuity, but further magnifying emotional load and cognitive fatigue. (Srivastava et al., 2024) have posited that Indian instructors felt less meaningfulness, psychological safety, and emotional availability with the abrupt transition to online schooling. Coping mechanisms were institutionally centered and on knowledge sharing; though these acted as buffer effects in de-catastrophizing, they did not necessarily stop the cognitive and affective dissonance accumulated in speeded digital migration.

Cognitive overload to the learner is the most important problem in collaborative and solitary e-learning settings. (Cheng et al., 2023), in their research with expectation confirmation theory, established that high student cognitive load decreased learner satisfaction and perceived utility of web-based systems, creating a feedback loop that minimized learner engagement. This is corroborated by (Lee et al., 2024), who, in an experiment, demonstrated that high-fidelity instruments such as real-time monitoring dashboards of student activity actually enable instructor cognitive overload and stress unless calibrated. This is proof that computer-based interventions to enhance teaching effectiveness could unwittingly shift burdens of cognition without adequately aligning users’ information processing capacity.

Technostress, as a cognate psychological phenomenon encompassing digital overload, insecurity, and fatigue, has also drawn increasing empirical interest in turn. (Asad et al., 2023), drawing on a behemoth study in Pakistan, validated the existence of very high relationships between technostressors—namely, techno-insecurity—and the psychological well-being of postgraduate students. By implication, their results point to differential sensitivity to technology-induced stress by gender and knowledge of areas, which is of concern with respect to digital uptake equity among learner subgroups. Information overload and uncertainty also exacerbate cognitive exhaustion in virtual environments. (Wei et al., 2025) illustrated how micro-video websites, which initially appear to be interactive, could potentially lead to user weariness and abandonment as a mechanism for cognitive load when it exceeds perceived content value. This is more common in learning environments where message intricacy, screen time, and interference with learning sessions lead to prolonged mental exhaustion and diminishing attention spans.

In addition, numerous studies have reported difficulties in cognitive and psychological equilibrium under flipped and blended learning paradigms. (Antoniadou et al., 2022) identified that dental students embraced e-learning for theoretical education but lost confidence regarding the mastering of practical skills, a cognitive dissonance in the methods of learning and content type. Similarly, (Kovtaniuk et al., 2025) noted the potential of resources like Canva to mitigate certain cognitive loads via the provision of aesthetically pleasing content; yet, these resources cannot fully remedy deeper issues regarding motivation and attentional fragmentation.

It is noteworthy that scholarship recognizes teachers’ psychological workload. (Gould et al., 2022) restated Cognitive Load Theory (CLT) as teacher-centered, calling for minimizing extraneous load through systematic digital pedagogy and teacher professional development. Their redefinition of CLT emphasizes the too-often-overlooked emotional labor that teachers need to perform in order to maintain an online “classroom presence” in the context of technologies and psychological burnout. Further global syntheses, such as the multi-national research by (Bozkurt et al., 2020), reproduce this result in reporting to what extent ERT exacerbated already existing digital divides and amplified emotional vulnerability amongst students, teachers, and families. The authors call for a pedagogy of care—founded on empathy and emotional attunement—as an unavoidable counter-trend to digital acceleration and its psychosocial consequences.

Taken together, these studies draw a subtle picture of psychological and cognitive dynamics in virtual learning environments. Virtual technology introduces novel affordances for scalable learning, but unobtrusive stressors are introduced that impact well-being, learning efficiency, and teaching effectiveness. A general failing across studies is a lack of adequate theorizing of how individual differences—personality, resilience, and digital self-efficacy—mediate reactions to cognitive load (Bozkurt et al., 2020; Cheng et al., 2023; Gould et al., 2022).

Moreover, few researchers put together psychological pressure with theory such as motivational autonomy or perceived control, leaving us without a proper appreciation of how students and teachers navigate mental requirements in coercive or gamified online learning environments. By integrating psychological pressure and cognitive load into its framework as critical components, this research suggests a more holistic approach to understanding EdTech persuasion and placing cognitive and affective costs centrally within user experience and system ethics.

### 2.3. Autonomy and Motivation in Smart Learning

After a flourishing body of interdisciplinary literature, the dynamics between autonomy, motivation, and wise learning environments have taken center stage in digital learning research (Coelho et al., 2025; Daniel et al., 2024; Dicheva et al., 2023; Fan, 2023). Central to this research is the use of Self-Determination Theory (SDT), which situates autonomy, competence, and relatedness as inherent psychological needs facilitating intrinsic motivation (Ismailov & Ono, 2021; Kian et al., 2022; Schmid & Schoop, 2022). Spanning various settings—from gamified learning systems to phone-based language learning apps—empirical research repeatedly confirms that pedagogic relevance and perceptions of learner autonomy are central to maintaining motivation and engagement in smart learning environments.

For example, (Terkaj et al., 2024) speak of the motivational effect of virtual learning factories in higher education. In their study, they suggest that students can be induced to explore authentic industrial situations through interactive immersive environments and, thus, promote perceived autonomy and interest. This is also elucidated by (Yu et al., 2023), who confirm that mobile English learners are much more motivated and have better learning achievement compared to traditional ones, and they state that autonomy-supportive mobile instruments promote strategic learning behavior and enhanced outcomes.

There are cumulative learning effects based on gamification research. (Dicheva et al., 2023) reveal the manner in which virtual currency within gamified settings induces motivation but does not induce intrinsic motivation or accomplishment in learning. On the other hand, (Kian et al., 2022) display how aspects of game features appealing to students’ psychological types, i.e., those triggering group pursuit, challenge, and time pressure, can internalize extrinsic rewards in terms of intrinsic motivation, especially when based on SDT and directed towards learner types. But, they also caution that poorly designed features like too much choice or questioning can overwhelm and dismantle engagement and require scaffolding and customization of e-pedagogy.

The tension between autonomy and instructional design is also present in comparative research comparing synchronous and asynchronous learning. (Zsifkovits et al., 2025) conclude that asynchronous modes are more socially accepted, but synchronous designs initiate more intrinsic motivation to a higher degree, being moderated by perceived autonomy. The tension between flexibility and interactive immediacy explains that autonomy is not so much a question of learner control, but of feeling psychologically free and capable within designed settings.

Greater complexity may be noted when discussing the emotional and motivational effect of distance learning during a crisis. (Ismailov & Ono, 2021) present that, in the pandemic situation of the COVID-19 pandemic, activities maximizing autonomy, personal meaning, and social interaction were optimal to maintain learners’ motivation and those perceived as too difficult or irrelevant harmed motivation. In the same line, (Suriagiri et al., 2022) conclude that, although digital technology creates greater enjoyment and interest, the traditional classroom setting still perpetuates greater autonomy and belongingness or high satisfaction. They believe that hybrid models can best serve the multiple needs of the learners.

Interpreting these findings, (Coelho et al., 2025) adopt an experimental approach in systematically testing the effects of badges, points, and challenges on motivation. While all gamified conditions enhanced learning compared to a disclosed control group, sole usage of badges caused cognitive overload without necessarily enhancing motivation. Their research substantiates the contention that autonomy-supporting design must balance feedback mechanisms against learners’ cognitive capacity and motivational needs. Finally, as a point of culmination, (Daniel et al., 2024), through their comprehensive systematic review, identify emergent pedagogies—flipped classrooms, virtual reality, and AI-supported feedback—as key enablers of blended learning performance and motivation. They emphasize the facilitator role of support in the creation of advanced self-regulated learning environments that are both technologically rich and pedagogically rich.

Collectively, these papers refer to the awareness that learner autonomy is both the outcome and mediator of effective smart learning environments. In whatever guise—via adaptive gamification, mobile frontends, or asynchronous collaboration—motivation is served best where digital affordances intersect the learners’ contextual requirements and their psychological requirements. But, they generate paradoxes—such as the possible benefits and tensions of interactivity at the same time—and need to be carefully designed based on evidence. In integrating autonomy, orientation, and motivational theory with affective and persuasive elements of EdTech, the present study contributes to this new discipline by providing an integrated model in attempting to satisfy cognitive, motivational, and emotional dynamics of technology-enhanced learning. To this end, the following hypotheses were formed:

**H1.**  *Perceived Persuasiveness of Platform Design (PPS) has a direct effect on Intrinsic Motivation for Learning (INTR).*

**H2.**  *Frequency of Nudge Exposure (NE) has a direct effect on Intrinsic Motivation for Learning (INTR).*

**H3.**  *Perceived Personalization (PP) has a direct effect on Intrinsic Motivation for Learning (INTR).*

**H4a.**  *Cognitive overload (COG) has a direct effect on Intrinsic Motivation for Learning (INTR).*

**H4b.**  *Perceived autonomy (PAUTO) has a direct effect on Intrinsic Motivation for Learning (INTR).*

**H5a.**  *Cognitive overload mediates the relationship between Perceived Persuasiveness of Platform Design and Intrinsic Motivation for Learning.*

**H5b.**  *Perceived autonomy mediates the relationship between Perceived Persuasiveness of Platform Design and Intrinsic Motivation for Learning.*

**H6a.**  *Cognitive overload mediates the relationship between Frequency of Nudge Exposure and Intrinsic Motivation for Learning.*

**H6b.**  *Perceived autonomy mediates the relationship between Frequency of Nudge Exposure and Intrinsic Motivation for Learning.*

**H7a.**  *Cognitive overload mediates the relationship between Perceived Personalization and Intrinsic Motivation for Learning.*

**H7b.**  *Perceived autonomy mediates the relationship between Perceived Personalization and Intrinsic Motivation for Learning.*

## 3. Methodology

### 3.1. Conceptual Model and Rationale

The heightened use of smart learning systems, like Moodle, Coursera, Duolingo, and Khan Academy, has revolutionized the learning experience by embracing persuasive design elements from behavioral science and digital marketing. Gamified badges, progress prompts, streaks, and personalized recommendations are designed to encourage ongoing use. Yet, whereas such affordances can optimize behavioral adherence, their psychological impact on learners is less clear. Recent research has contended that such persuasive interfaces can operate less as motivational support and more as tools of subtle control, eroding learner autonomy and adding to cognitive load (Aldenaini et al., 2020; Asad et al., 2023; Chapman et al., 2025).

Operationally, we handle EdTech nudges as interface-level defaults and prompts presented within learning episodes (NE), not as a more general persuasive design tone (PPS), and to tailor content/feedback (PP). This is the distinction that makes it clear that the nudges are the micro-level delivery mechanism, PPS encapsulates the macro-level persuasiveness of the entire interface, and PP encapsulates the content fit with the learner. With this gap, current studies develop and test a conceptual model based on Self-Determination Theory (SDT), Cognitive Load Theory, and knowledge of Persuasive Systems Design (Bosch, 2024; Cheng et al., 2023; Coelho et al., 2025). The model explores how perceived platform design persuasiveness and Nudge Exposure frequency influence students’ intrinsic motivation, and psychological pressure and perceived autonomy serve as mediators between them. The research also examines the moderating effect of autonomy orientation, a trait-level characteristic that indicates the degree to which individuals are self-regulated in making their choices (Brown et al., 2023; Choi & Lee, 2022; De Ridder et al., 2022).

Perceived persuasiveness is the degree to which the platform interface, its functionality, and its messages are perceived by the learners to be intended to shape their behavior. Persuasive interface design, particularly if it is founded on iterative nudging, gamification, and reward mechanisms, can potentially flip the learner experience from being one of agency to manipulation. Previous research on persuasive technology and on HCI has also identified that highly persuasive systems can undermine people’s volitional control. In schooling, it could be in the form of heightened psychological pressure—felt as internal tension, mental workload, or performance anxiety—particularly when learners feel they have to sustain streaks, gain points, or react to prompts (Chapman et al., 2025; Cheng et al., 2023; Cheung & Rogut, 2024). These conditions are, in SDT’s view, controlling and inversely related to optimal motivation.

At the same time, regular exposure to nudges, meaning small prompts or reminders aimed at nudging action, can also be responsible for this feeling of pressure. While nudges are a reminder or spur, inordinate or untimely nudging has been revealed to enhance extraneous cognitive load as well as affective fatigue (Choi & Lee, 2022; Coelho et al., 2025; Das et al., 2025). In intelligent learning environments, students can be exposed over and over again to stimuli intended to prompt action, which, if viewed as intrusions, create mental overload and reactance. This is in line with results from digital media and advertising scholarship, whereby message repetition and behavior targeting have been linked to reactance and disengagement (Daniel et al., 2024; De Ridder et al., 2022; Dicheva et al., 2023).

Psychological pressure can be defined as the mediating process through which nudge frequency and perceived persuasiveness affect motivational outcomes. SDT argues that, when they believe to be externally controlled or pressured, intrinsic motivation dwindles. Rather than doing something because they are interested or enjoy it, students might start doing it because they feel forced or to avoid punishment. This research also examines the indirect effect of psychological pressure on the perception of autonomy, the basic psychological need of SDT (Lee et al., 2024; F. A. Orji et al., 2024; Plak et al., 2023). With the mounting pressure, students begin to feel less control over their learning process, which further erodes intrinsic motivation.

Conversely, perceived autonomy is a protection mechanism and a second mediator of the model. Learning environments that facilitate learner autonomy—by providing choice, flexibility, and purposeful learning trajectories—are always linked with increased intrinsic motivation and motivation (Knutas et al., 2019; Martin & Kwaku, 2019; McCarthy et al., 2024). As long as students have a sense of control over their options, even with coercive signs, their intrinsic motivation will be better protected. Therefore, autonomy not only serves as a direct motivator, but also as an avenue through which platform design features have an effect (Chapman et al., 2025; Cheung & Rogut, 2024).

Lastly, autonomy orientation is postulated as a moderator, measuring variation in individuals in self-regulating and resisting control. High-autonomy-oriented students are hypothesized to be less susceptible to persuasive influence, feeling less psychological pressure and having more intrinsic motivation even when exposed to nudges. In contrast, low-autonomy-oriented people are more likely to be prone to influence cues and have higher pressure responses. Trait-level moderators such as this one have been found to have considerable influence on user experience in persuasive technology and e-learning studies (Daniel et al., 2024; Das et al., 2025).

**Figure 1:**
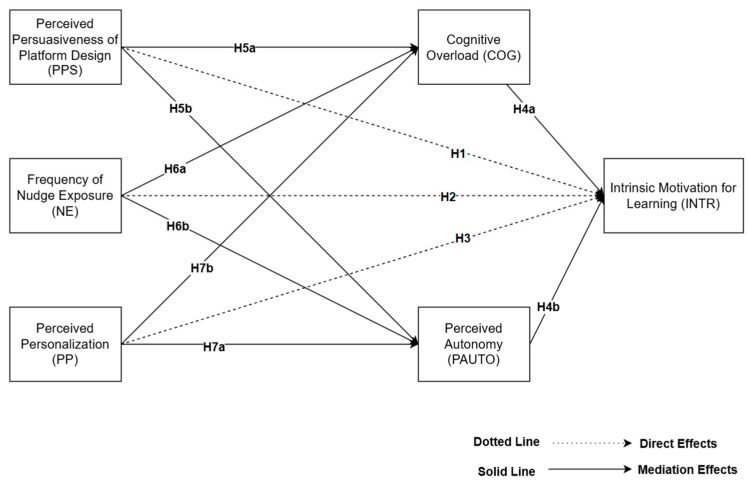
Conceptual model.

Overall, the model is of theoretical and practical merit. Theoretically, it is an addition to SDT and persuasive design scholarship in that it marks out the two paths—of pressure and autonomy—through which persuasive EdTech can influence motivation. Practically, it offers evidence that can be used in informing the ethical design of smart learning systems so that engagement is not antithetical to learner well-being and agency. This integrated process is relevant since learning environments increasingly depend on marketing-based approaches for the purpose of influencing learner behavior. Figure 1 illustrates the conceptual model employed during this study, illustrating hypothesized relationships among variables of interest.

### 3.2. Data Collection and Sampling

In order to examine the effects of persuasive design elements in intelligent learning environments on psychological pressure and intrinsic motivation in students at a university, a cross-sectional questionnaire survey was conducted (Kesmodel, 2018; Olsen & St George, 2004). A non-probability convenience sampling strategy supplemented by snowball sampling was employed to achieve a heterogeneous sample of students with recent exposure to online learning situations (Rahman, 2023; Rahman et al., 2022). The use of this strategy was appropriate considering the behavioral modeling goals of the study and inclusion criteria that were relevant to the use of persuasive learning technologies in the last three months. Convenience sampling facilitated easy access to participants through institutional communication channels, while snowball recruitment facilitated reach extending over a greater breadth across disciplines and study levels and enhancing the heterogeneity and ecological validity of the dataset (Sandelowski, 2000; Spector, 2019).

Data were gathered through an online survey created using Google Forms that had three parts: (1) screening and informed consent, (2) demographic and platform use information, and (3) psychometric instruments assessing the study’s main constructs. The survey utilized validated tools from previous studies in educational psychology, persuasive technology, and Self-Determination Theory. These were persuasiveness of platform design, exposure frequency of nudge, psychological pressure, perceived autonomy, intrinsic motivation, and trait-level autonomy orientation. Pilot testing of the questionnaire was conducted on a sample of 20 university students for testing clarity, face validity, and content relevance to the research setting. Minor revisions were made based on pilot feedback, including clarifying item wording that referenced platform-specific features (e.g., badges, pop-up reminders).

Participants were eligible to take part if they fulfilled the following inclusion criteria: (a) 18 years or older; (b) studying a course of higher education (Bachelor’s, Master’s, or PhD); and (c) had used at least one smart learning platform (e.g., Moodle, Coursera, Duolingo, Khan Academy, or YouTube for education) during the previous three months. These inclusion standards guaranteed that participants were able to provide informed consent and had experienced recent appropriate use of digital platforms with persuasive elements, the provision of which is necessary to measure the constructs of interest. The exclusion criteria were (a) those younger than 18 years; (b) participants not enrolled in any formal study program; (c) participants with no recent experience of learning platforms; and (d) incomplete or screening failure responses. Screening questions integrated into the start of the survey automatically excluded ineligible individuals to maintain harmony with the study’s scope and internal validity.

The last survey was released online through university mailing lists, departmental LMS web sites (e.g., Moodle), and student social media networks for four weeks. It was anonymous and voluntary. Before they could access the full questionnaire, all respondents had to read and sign an informed consent form explaining the purpose, steps, data privacy protections, and their right to withdraw at any time without penalty.

A total of 740 usable responses were kept for analysis. The minimum sample was calculated from Structural Equation Modeling (SEM) best practice, which recommends a ratio of participants to parameters of at least 10:1. Considering the model in hand to be complex, with more than one latent construct and mediation/moderation effects included in it, a minimum sample of 400 participants was set, as recommended by (Hair et al., 2021a; Memon et al., 2020; Wagner & Grimm, 2023). This sample size was deemed adequate to provide stable model estimation, detect medium effect sizes, and to allow subgroup analysis by Multi-Group Analysis (MGA) for major demographic and behavior factors (Memon et al., 2020; Van Zyl & Ten Klooster, 2022).

For ensuring validity and reliability, the measuring items were all sourced from psychometrically supported scales and minimally adapted only to suit the smart learning environment. Internal construct consistency with over one item was estimated with Cronbach’s alpha, and scores ≥ 0.70 were employed. Two experts in the domains of educational technology and behavioral science reviewed the items of the questionnaire for semantic accuracy and construct validity. In the final instrument, latent construct items employed 5-point Likert scales, while demographic and platform use questions were closed-ended (multiple- or single-choice) items. There were no open-ended items. All psychometric items were borrowed from established scales in the literature (see Section 3.3) and were inspected by the two domain experts and pilot-tested for validity and contextual fit. Ethical issues were dealt with strictly according to GDPR and ethics guidelines of the university. The survey was completely anonymous, and no personal data identifiable to individuals were gathered. Contributors were clearly made aware of their rights and that contributing was voluntary. There were no vulnerable individuals involved in the study, nor was deception or risk-inducing material employed.

### 3.3. Measurement Scales

All latent scales were assessed with items taken from validated scales and worded minimally to the smart learning environment used and modified for context application in pedagogically stimulating technologies (see Table A1, Appendix A). Adaptation was to surface wording (e.g., badge name, reminder, progress bar), preserving original constructs’ meaning and factor structure. Likert anchors were from 1 = strongly disagree to 5 = strongly agree and applied solely to the psychometric items; demographic and usage items were categorical. As reported by the findings, NE5 and PP5 were excluded after standard reliability/validity diagnostics. The Perceived Persuasiveness of Platform Design factor was measured using five items that captured the extent to which students perceived the persuasive purpose being integrated into platform features, including badges, reminders, and progress bars (e.g., “The platform encourages me to keep going even when I don’t intend to”) (Thomas et al., 2019). Frequency of Nudge Exposure (NE) was assessed using four items that asked the participants how often they were experiencing nudging features while they utilized the platform (e.g., “The platform often asks me to do extra things”) (Brick et al., 2023). The fourth item was not included in pretesting because it was a duplicate. Perceived Personalization (PP) was evaluated with four items that probed to what degree students felt the content, feedback, and advice were suited to their individual needs and learning path (e.g., “The lessons or advice are suited to my progress”) (Aguirre et al., 2015). One item was dropped for the sake of internal consistency. Cognitive Overload (COG) was assessed by three items describing the psychological load experienced by the users as a response to system requirements and expectations (e.g., “I feel stressed when I do not live up to the system’s expectations”) (Choi & Lee, 2022). Perceived autonomy (PAUTO) was assessed by three items describing the extent to which the learners felt they were volitional and self-controlled when interacting with the platform (e.g., “My learning process feels self-initiated, and not imposed on me”) (Bosch, 2024). Lastly, Intrinsic Motivation for Learning (INTR) was assessed through three items that tapped participants’ self-report of interest, enjoyment, and willingness to use the system again (e.g., “Learning with this system is fun and rewarding”). All the items were examined through confirmatory factor analysis for construct validity and Cronbach’s alpha, and composite reliability indices were used to estimate reliability.

### 3.4. Sample Profile

A total of 740 participants were recruited in this study (Table 1). The sample was equally distributed in terms of gender, with 374 females (50.5%) and 366 males (49.5%). Most of the respondents belonged to the age group of 21–23 years (25.9%), followed by 27–29 years (22.2%), 24–26 years (21.8%), 30+ years (15.4%), and 18–20 years (14.7%). Educationally, 33.1% of the respondents were existing undergraduate students, 28.2% had a bachelor’s degree, and 23.9% had a master’s degree or higher. A lower percentage (14.7%) reported a high school diploma as the highest level of education. As for how often they used learning platforms, 24.9% said they used platforms occasionally or when necessary, while others used platforms a few times a month (21.6%), once per week (15.4%), a few times per week (17.4%), or daily (20.7%). Some of the respondents were more knowledgeable than others about persuasive elements on learning platforms. Close to 28.0% said that they were very familiar and 23.8% were relatively familiar with such aspects. On the other hand, 18.6% were not very familiar and 29.6% were not familiar at all. When asked what device they used most to access learning platforms, 24.1% said desktop computer, followed by laptop (21.4%), smartphone (21.1%), other devices (21.9%), and tablet (11.6%). Self-assessed digital literacy was generally high, with 47.6% of respondents reporting having very high confidence, 17.4% having high, and 6.2% having moderate. But, 12.2% had low and 16.6% had very low digital literacy. Utilization of smart learning platforms within the last three months (multiple response) had YouTube as most utilized (22.8%), followed by Khan Academy (18.9%), Moodle (18.1%), Other platforms (18.2%), Coursera (15.0%), and Duolingo (6.9%).

## 4. Data Analysis and Results

The structural analysis of the research was achieved through the use of the Structural Equation Modeling (SEM) approach with the support of SmartPLS 4 (Version 4.1.1.4). Adopting the approach presented by (Nitzl et al., 2016), SEM—its variance-based variant—is generally recognized as a sound method of analysis for studies in the field of management and social sciences. Partial Least Squares Structural Equation Modeling (PLS-SEM) was used because it has the capability to estimate causality and maximize explained variance in endogenous latent variables (Cheah et al., 2023; Matthews, 2017; Sarstedt et al., 2011). Multi-Group Analysis (MGA) was utilized in studying subgroup differences for finding contextual differences in structural relations that otherwise could not be observed with conventional regression techniques (Cheah et al., 2023; Matthews, 2017; Sarstedt et al., 2011). The estimation procedure followed (Wong, 2013) guidelines to ensure the firm calculation of path coefficients, standard errors, and reliability estimates. In the reflective measurement model, outer loadings were checked for indicator reliability, and loadings greater than 0.70 were satisfactory.

### 4.1. Common Method Bias (CMB)

In order to cross-validate the validity and reliability of findings, a systematic CMB analysis was conducted as per the procedural guidelines of (Podsakoff et al., 2003). For this, Harman’s single-factor test was conducted in order to verify whether one latent factor was contributing most of the variance between items. The unrotated principal component analysis indicated that the largest component accounted for 35,114% of total variance, far lower than the commonly employed 50% cut-off. While CMB did not emerge as a key risk in the present study, the inclusion of CMB enhances the validity of the reported associations and enhances assurance of the robustness of findings by correcting for possible sources of bias (Podsakoff et al., 2003, 2012).

### 4.2. Measurement Model

The first step of the PLS-SEM procedure is the assessment of the measurement model, in which all the constructs are operationalized via reflective indicators. Following the criteria suggested by (Hair et al., 2014), such an assessment is based on four major criteria: composite reliability, indicator reliability, convergent validity, and discriminant validity.

The first step in PLS-SEM is to test the reflective measurement model (Hair et al., 2014) based upon four criteria: composite reliability, indicator reliability, convergent validity, and discriminant validity. Indicator reliability is a percentage of variance in an indicator caused by its latent construct and is estimated based upon outer loadings. Values ≥ 0.70 are preferred (Wong, 2013; Chin, 1998), while, in social sciences, applications of lower values around 0.70 would be acceptable if its deletion does not adversely impact validity/reliability in a construct (Vinzi et al., 2010). As advocated by (Hair et al., 2014), such indicators around 0.40–0.70 should only be deleted if their deletion considerably enhances composite reliability or AVE. Guided by these rules, two indicators—NE5 and PP5—were removed from the final model since their loadings were less than the 0.500 cut-off point. This process, guided by the criteria developed by (Gefen & Straub, 2005), was intended to increase the quality and reliability of the measurement model as a whole (see Table 2).

This table shows each item’s outer factor loadings on its respective latent construct, along with the internal consistency measures: Cronbach’s alpha, rho_A, composite reliability (CR), and average variance extracted (AVE). Loadings greater than 0.70 are acceptable.

Measurement model reliability was confirmed by Cronbach’s alpha, rho_A, and composite reliability measures. A minimum of 0.70 that (Wasko & Faraj, 2005) suggested was attained for such dimensions as cognitive overload (COG), intrinsic motivation (INTR), Nudge Exposure (NE), perceived autonomy (PAUTO), Perceived Personalization (PP), and perceived persuasiveness (PPS). The other constructs also had good-to-excellent reliability, according to findings in earlier work (Hair et al., 2006; Lam, 2012; Pervan et al., 2017). The rho_A coefficient, theoretically ranging between Cronbach’s alpha and composite reliability, was more than 0.70 for most of the constructs, as suggested by reliability standards dictated by (Sarstedt et al., 2021) and reiterated by (Henseler et al., 2015).

Convergent validity was similarly considered to be acceptable, in that the average variance extracted (AVE) was above 0.50 in the majority of the constructs following the requirements of (Fornell & Larcker, 1981). Where AVE measures were slightly below 0.50, validity was also accepted where respective composite reliability was above 0.60, since (Fornell & Larcker, 1981) also took this argument into consideration. Discriminant validity was also assured by the inter-construct correlation analysis with correlations being lower than the square root of the AVE for all the scales, hence meeting the Fornell–Larcker criterion. Further validation was through the heterotrait–monotrait (HTMT) ratio of correlations as suggested by (Henseler et al., 2015), such that all the HTMT were below the conservative cut-off value of 0.85 (see Table 3 and Table 4).

### 4.3. Structural Model

The structural model was assessed by checking the coefficient of determination (R^2^), predictive relevance (Q^2^), and path coefficients’ significance, following procedures outlined by (Hair et al., 2011). The R^2^ of the endogenous constructs was 0.472 (cognitive overload (COG)), 0.481 (intrinsic motivation (INTR)), and 0.468 (perceived autonomy (PAUTO)), reflecting moderate explanatory power within the acceptable range between 0 and 1. Predictive relevance, as assessed by Q^2^, also reflected moderate-to-strong predictive accuracy, with values of 0.465 (COG), 0.432 (INTR), and 0.461 (PAUTO). The structural relationships between constructs were also confirmed using hypothesis testing. The path weights were estimated using the non-parametric bootstrapping procedure according to the recommendations of (Hair et al., 2021b). The mediation effects were examined with a bias-corrected bootstrapping approach, using 10,000 resamples, following the procedure outlined by (Preacher & Hayes, 2008) and later explicated by (Streukens & Leroi-Werelds, 2016). A summary of the mediation effects and path coefficients is shown in Table 5.

Structural model results gave strong empirical support for all tested direct hypotheses, revealing that the core persuasive and psychological variables are significant to influence students’ intrinsic motivation in online learning contexts. In particular, Perceived Persuasiveness of Platform Design (PPS) positively and significantly influenced intrinsic motivation (INTR) (β = 0.118, t = 3.606, *p* < 0.001), showing that the more students view the design features of an education platform—e.g., gamified badges, visualized progress, or interactive nudges—as persuasive and meaningful, the more they will internalize motivation to learn. This aligns with prior work in persuasive system design on the motivational strength of user interface cues (Huang et al., 2024). Exposure to Nudges (NE) also strongly predicted intrinsic motivation (β = 0.211, t = 6.250, *p* < 0.001), showing that the greater the exposure of a student to such behavioral prompts—e.g., streak reminders, leaderboard ranking, or invites to keep learning—the higher the intrinsic motivation is. This affirms the argument that nudges have the capability to support learning behavior through constant reinforcement without necessarily infringing on autonomy, especially when learners feel they are assisted but not controlled. Perceived Personalization (PP) emerged as the strongest direct predictor of intrinsic motivation (β = 0.273, t = 6.268, *p* < 0.001).

This result underscores the key position played by content adaptation, adaptive feedback, and contextual salience to promote engagement and self-paced learning. If users perceive that the learning environment is attuned to their goals, preferences, or earlier conduct, they will find enjoyment, relevance, and persistence, pillars of Self-Determination-Theory-based intrinsic motivation (Huang et al., 2024). Most notably, perhaps, cognitive overload (COG) also positively correlated, albeit weakly, with intrinsic motivation (β = 0.096, t = 2.474, *p* = 0.007), a result which, at first sight, might be considered paradoxical. A likely explanation is that properly controlled amounts of challenge or informational density can induce curiosity and interest, a process in line with the “desirable difficulty” effect in the psychology of learning. But, the impact should be interpreted with care, since excessive cognitive load might, in various contexts, depress motivation. Finally, perceived autonomy (PAUTO) also arrived with a robust and significant positive impact on intrinsic motivation (β = 0.267, t = 6.948, *p* < 0.001), as expected, substantiating the root significance of generating learners’ sense of volition, control, and choice. This is consistent with much of the Self-Determination Theory literature, which takes autonomy as a fundamental psychological need driving intrinsic motivation. In combination, these findings suggest that motivational educational systems must strike a balance between effective nudging, personalization, and autonomy support in order to foster the optimal learner engagement. Results are theory-driven for integrating aspects of persuasive system design with motivational psychology and provide design recommendations for crafting online learning environments that transcend compliance and move toward actual learner empowerment.

### 4.4. Mediation Analysis

To explore the psychological processes by which design- and persuasion-based platform features impact learners’ intrinsic motivation, mediation analyses were performed in succession. Cognitive overload (COG) and perceived autonomy (PAUTO) were of particular interest as mediators of the effect of three independent variables—Perceived Persuasiveness of Platform Design (PPS), Frequency of Nudge Exposure (NE), and Perceived Personalization (PP)—on the dependent variable, Intrinsic Motivation for Learning (INTR). Bootstrapping methods with 10,000 bias-corrected resamples were used to estimate direct and indirect effect significance (Table 6).

All three predictors exerted statistically significant direct effects on intrinsic motivation. As hypothesized (H1), PPS was directly positively and significantly related to INTR (β = 0.118, SE = 0.033, t = 3.606, *p* < 0.001), which indicates that gamified visualizations and motivational cues such as persuasive elements can improve students’ engagement. NE also directly affected INTR to a large extent (H2; β = 0.211, SE = 0.034, t = 6.250, *p* < 0.001), i.e., repeated exposure to behaviorally nudged material leads to states of intrinsic motivation that last. In the same manner, PP exerted the most powerful direct effect (H3; β = 0.273, SE = 0.044, t = 6.268, *p* < 0.001), which underscores how adaptive platform experience and customized content play a key role in ensuring intrinsic motivation.

The test of indirect effect revealed heterogeneous mediation effects. For PPS, the indirect effects through the mediators were both negative and statistically significant. That is, there was an indirect effect of PPS on INTR through COG (H5a; β = −0.013, SE = 0.005, t = 2.464, *p* = 0.007) and through PAUTO (H5b; β = −0.045, SE = 0.011, t = 4.137, *p* < 0.001). This is because, while PPS boosts motivation in a direct manner, it also boosts cognitive overload and lowers users’ perceived autonomy, factors that erode intrinsic motivation. As the direct effect and indirect effect are of opposing signs, this pattern suggests competitive partial mediation. These findings are consistent with theoretical worry from Cognitive Load Theory and Self-Determination Theory that very directive or very persuasive technology will engage individuals in the short run but only at a cost to their sense of agency and cognitive coherence (Kawa et al., 2023; Kian et al., 2022).

Conversely, NE’s mediation model indicated complementary partial mediation. NE recorded significant cognitive overload decreases (H6a; β = 0.021, SE = 0.010, t = 2.155, *p* = 0.016) and increased perceived autonomy (H6b; β = 0.028, SE = 0.011, t = 2.555, *p* = 0.005), both of which were significantly associated with INTR. These secondary influences complemented the positive direct impact of NE, which hypothesized that nudges, placed in a supportive rather than coercive frame, increase intrinsic motivation through maximizing cognitive and psychological states. This complementary mediation pattern is consistent with more recent conceptions of digital nudging as an apparatus that can be internalized into autonomous self-regulation so long as they are employed ethically and transparently. The largest indirect effects were in PP’s case, and it also exhibited complementary partial mediation. Both of the paths—PP → COG → INTR (H7a; β = 0.048, SE = 0.019, t = 2.485, *p* = 0.006) and PP → PAUTO → INTR (H7b; β = 0.154, SE = 0.022, t = 6.917, *p* < 0.001)—were positive and significant statistically, supporting the expectation that personalization not only does so through content fit, but also decreases mental effort and increases perceived volition. This outcome is consistent with Self-Determination Theory and Expectancy–Value Theory, which both highlight the motivational benefits of autonomy-supportive significance-matched learning environments.

Parallel to this, findings show that platform features have both positive and negative impacts on students’ intrinsic motivation. Nudging (NE) and personalization (PP) showed complementary mediation where motivation was enhanced directly and indirectly through reduced cognitive overload and higher volition. Persuasive design (PPS), however, exhibited competitive partial mediation where motivational gain was offset by a partial imposition of psychological costs such as cognitive burden and lower volition. These findings imply that argumentative strategies should be used carefully in instructional technology development, balancing users’ cognitive abilities and autonomy. Educators and interface designers are invited to reduce intransitive or controlling cues, rather than using personalization and encouraging nudges, in order to maximize learner motivation and well-being.

### 4.5. Multi-Group Analysis (MGA)

To explore the moderating effect of influential user traits on the structural model, Multi-Group Analyses (MGAs) with two-tailed significance tests were carried out in SmartPLS. Substantially varying path coefficients between subgroups point towards a decisive influence of user traits on the impact of digital persuasion mechanisms on cognitive and motivational consequences (see Table 7).

#### 4.5.1. Gender-Based Group Differences

The tests for the MGA indicated that gender strongly interacted with most of the structural paths in the model. Cognitive overload (COG) had a much stronger effect on intrinsic motivation (INTR) for male participants compared to female participants (Δβ = 0.427, *p* < 0.001), and this implies that cognitive tension could have a greater motivational function in men. Likewise, Perceived Persuasiveness of Platform Design (PPS)’s impact on INTR was also more pronounced in males (Δβ = 0.437, *p* < 0.001), which reflects higher sensitivity to persuasive platform-level design. Comparatively, women evidenced greater effects of Nudge Exposure Frequency (NE) on INTR (Δβ = −0.274, *p* < 0.001) and of Perceived Personalization (PP) on INTR (Δβ = −0.306, *p* < 0.001), which implies that emotionally supportive factors and personalized features are more powerful in stimulating learning motivation among women. There were other gender differences in mediation pathways. PPS had a much greater influence on COG in men (Δβ = 0.200, *p* = 0.006), whereas NE → PAUTO was greater in women (Δβ = −0.181, *p* = 0.008). A marginally significant contrast was also found for the PP → PAUTO path (Δβ = 0.104, *p* = 0.051), with slightly larger autonomy from tailored content for men. These trends highlight the need for gender-sensitive persuasive design, where males can be supported by mental stimulation and persuasiveness throughout the system and females can be encouraged by affective support and personalization through autonomy.

#### 4.5.2. Age-Based Group Differences

Age also moderated several model pathways. The NE → PAUTO pathway was particularly stronger in the youngest students between 18 and 20 years compared to older groups, and this indicates that the effect of behavioral nudges on subjective autonomy is more pervasive in early-stage learners. The PP → INTR pathway, conversely, was weaker for the youngest group and stronger in older students and captures variation in development in how personalization affects motivation. Younger participants also experienced more negative cognitive impacts from nudging and personalization (NE → COG and PP → COG), while older participants were less susceptible to adaptive elements without suffering from more cognitive load. Persuasive design (PPS) also had varying impacts on both COG and PAUTO across age category, with the oldest participants (27–29) experiencing fewer instances of cognitive overload and more autonomy. Finally, the COG → INTR and PAUTO → INTR patterns also differed, showing age-differentiated differences in the control of motivation by cognitive or self-determined processes. In general, these findings indicate that persuasive and adaptive roles must be differently set by age to correspond to learners’ development and metacognitive competencies.

#### 4.5.3. Education-Level Group Differences

Education level substantially moderated different model paths, clarifying that the cognitive and motivational responses to platform features are also heterogeneous depending on educational background. The relationship between PP and PAUTO was stronger for Bachelor graduates than for High School graduates (Δβ = 0.294, *p* = 0.002) and even slightly stronger than for Undergraduate students (Δβ = −0.185, *p* = 0.035). Moreover, Bachelor’s students also reported greater PPS → PAUTO than High School students (Δβ = 0.302, *p* = 0.005), and Master’s students reported a greater degree of PPS → PAUTO than Undergraduate students (Δβ = −0.499, *p* = 0.005), indicating individuals with greater academic education find persuasive influences to be autonomy-promoting. Bachelor graduates were more sensitive to cognitive overload due to personalization (PP → COG, Δβ = 0.218, *p* = 0.014), and NE functioned more strongly to reduce COG for this segment (Δβ = −0.162, *p* = 0.044). In addition, the PPS → INTR pathway was preferable to individuals who had obtained Master’s degrees compared to High School graduates (Δβ = 0.256, *p* = 0.029), indicating higher motivational internalization of the persuasive design among more educated individuals. Finally, PAUTO → INTR was considerably greater in Master’s degree participants compared to High School diploma (Δβ = 0.268, *p* = 0.007) and Undergraduate participants (Δβ = 0.211, *p* = 0.017), indicating support for higher importance of autonomy in motivation in better-educated students.

#### 4.5.4. Digital-Literacy-Based Group Differences

Participants’ digital literacy also mediated core relationships. COG’s effect on INTR was significantly more significant for less digitally literate individuals (Δβ = −0.253, *p* < 0.001) in the sense that cognitive strain is a more effective deterrent to motivation when digital fluency is scarce. In contrast, PP had a greater positive effect on both PAUTO (Δβ = 0.267, *p* < 0.001) and INTR (Δβ = 0.469, *p* < 0.001) in high-literacy users, meaning that personalization is most effective when the users are able to properly understand and take advantage of personalized functions. NE had a larger negative impact on PAUTO (Δβ = −0.250, *p* = 0.001) and INTR (Δβ = −0.131, *p* = 0.022) in lower-literacy participants, perhaps by over-nudging or dissonance messaging. It is of interest that the PAUTO → INTR pathway was larger in high-literacy participants (Δβ = −0.207, *p* = 0.003), and this provides additional evidence for the hypothesis that self-directed learning is most productive when high technical competence prevails. Lastly, personalization was more cognitively effortful for high-literacy users (PP → COG, Δβ = 0.137, *p* = 0.037), perhaps because they engaged with system content in a more critical way.

#### 4.5.5. Familiarity with Persuasive Features

Moderating effects were also seen depending on participants’ experience with persuasive design. The cognitive overload effect on intrinsic motivation (COG → INTR) was much stronger for non-experienced participants in persuasive elements (Δβ = −0.310, *p* < 0.001), and so was the autonomy effect (PAUTO → INTR; Δβ = −0.285, *p* < 0.001). This would mean that non-experienced users feel more extreme negative or dependency-based effects. By contrast, older participants with influencing factors showed higher positive effects of NE on PAUTO (Δβ = 0.291, *p* < 0.001) and INTR (Δβ = 0.236, *p* = 0.001) and higher PP → INTR (Δβ = 0.166, *p* = 0.037). Interestingly, PPS → INTR was higher in users who were unaware of them (Δβ = −0.261, *p* = 0.001), indicating that users who are unaware are more susceptible to persuasive cues and less likely to respond in a more critically aware or adaptive way.

#### 4.5.6. Frequency of Platform Use

Last but not least, frequency of use also mediated model relations. NE → INTR was significantly stronger among frequent users (Δβ = 0.390, *p* < 0.001), indicating that nudging is increasingly motivational with increased familiarity with the platform. Conversely, PAUTO → INTR was stronger among infrequent users (Δβ = −0.330, *p* < 0.001), suggesting that autonomy is a more important source of motivation under conditions of infrequent use. Likewise, PPS → INTR (Δβ = −0.184, *p* = 0.005) and COG → INTR (Δβ = −0.194, *p* = 0.006) were stronger for low-frequency users, suggesting higher susceptibility to persuasive or cognitive load effects. Further differences were seen in the personalization and persuasion routes influencing cognitive overload: PP → COG and PPS → COG were stronger for infrequent users (Δβ = −0.135, *p* = 0.025 and Δβ = −0.135, *p* = 0.038, respectively). This indicates that less frequent users might feel more cognitive effort when dealing with persuasive and adaptive functions. A marginal difference in PAUTO → PPS (Δβ = 0.106, *p* = 0.052) was also found, suggesting a trend that more frequent users find persuasive design more autonomy-supportive.

Together, these subgroup analyses underscore the multidimensional and context-dependent character of persuasive and motivational processes in online learning. Optimized engagement effects require tailored, persuasive, and autonomy-supportive factors to be tuned to users’ demographic, cognitive, and experiential profiles.

## 5. Discussion

### 5.1. Direct Relationships: Interpreting the Effects of Platform Persuasion, Personalization, and Psychological States on Intrinsic Motivation

The structural model provided robust empirical support for the hypothesized direct effects, underscoring the relevance of persuasive system design and motivational theory in shaping learner engagement within educational technology environments. All five direct hypotheses (H1–H4b) were statistically supported, offering a nuanced understanding of how specific design elements and psychological experiences predict intrinsic motivation in online learning contexts.

Perceived Persuasiveness of Platform Design (PPS) was positively and significantly related to intrinsic motivation (INTR), suggesting that students who perceive the platform’s persuasive aspects, e.g., badges, progress visualizations, or reminders, as credible and meaningful are more inclined towards internalizing learning goals. This is in accordance with previous studies by (Huang et al., 2024), which revealed that the psychological perception of persuasiveness is responsible for mediating the effect of gamified factors on user behavior. Notably, this effect corroborates Self-Determination Theory’s (SDT) hypothesis that external contingencies may be internalized as intrinsic motivation if they are perceived as autonomy-supportive and non-controlling in nature (Martin & Kwaku, 2019; Murillo-Muñoz et al., 2021). Thus, persuasive messages can not only be cues to behavior, but be motivational aids if they are constructed with clarity, credibility, and user compatibility.

Exposure to Nudges (NE) was significantly positively related to intrinsic motivation. What this indicates is that multiple exposures to platform nudges—through the notification of streaks, reminders for progress, or leaderboard exposure—can activate a learner’s internalized motivation to learn. Though there has been criticism of nudges in the literature on the basis of loss of autonomy (Wiafe et al., 2024; Za et al., 2022), this current research demonstrates that the intervention becomes more powerful for intrinsic motivation if participants perceive it as assistance rather than coercion. This is the same result as from (Murillo-Muñoz et al., 2021), who demonstrated that nudging could be performed in education if designed and timed according to the expectations and needs of students. Design-wise, this means that platform nudges must be tailored, subtle, and even dismissible in an attempt not to cause reactance or overload.

Perceived Personalization (PP) was the most significant direct predictor of intrinsic motivation, confirming the central role of adaptive learning experience on increased learner engagement. This is supported by earlier studies by (Alslaity et al., 2023) and (F. A. Orji et al., 2024), which noted the impact of personalized persuasive strategies on increased user satisfaction and motivational consistency. Since students experience the content, feedback, and capability of a platform in a way they actually need for learning, they are going to feel the experience as purposeful and autonomous. Theoretically, this concurs with SDT, which assumes relevance and personal congruence as the basis of autonomy satisfaction. Pedagogically, these findings confirm the necessity for integrating real-time-adaptive and user-driven design on EdTech platforms to be able to maintain motivational engagement.

Counter to the trend, cognitive overload (COG) was positively and significantly related to intrinsic motivation, with a small effect size. While cognitive overload is traditionally argued to be harmful to learning, this finding might be indexing an “aptly difficulty” effect where moderate cognitive load produces deeper processing and engagement (Cheng et al., 2023). Or, maybe more plausibly, it is the case that very motivated students are demotivated by complexity but not by constraint. This explanation must be handled with care (Asad et al., 2023; Cheng et al., 2023; Gould et al., 2022). While the correlation was very large in magnitude, its size was quite modest, and in other classroom environments or with less dynamic students, information overload or choice overload will diminish motivation and well-being. Follow-up studies must examine this link longitudinally and across different levels of learner resilience and digital literacy (Murillo-Muñoz et al., 2021).

Lastly, perceived autonomy (PAUTO) had a significant and large positive influence on intrinsic motivation, consistent with its strategic position at the epicenter of student motivation. This is consistent with many decades of SDT-guided research, which positions autonomy as an invariant psychological need that needs to be fulfilled if students are to remain intrinsically motivated. In systems based on influence learning, this result will likely favor the argument that digital interventions need to be supportive of behavioral outcomes but also deferential to students’ sense of volition, autonomy, and freedom (Kian et al., 2022; Schmid & Schoop, 2022; Suriagiri et al., 2022). Even the most elegantly conceived nudges or gamification mechanisms can potentially boomerang if they are perceived as volition-, autonomy-, and freedom-toxic. The outcome confirms previous criticism of over-platform-control and underscores the significance of opt-in design, user agency, and responsive learning paths in effective EdTech design (Dicheva et al., 2023; Kian et al., 2022; Stein et al., 2024).

Taken together, these direct effects provide empirical rationale for an empirically balanced approach to persuasive learning systems, one favoring personalization, autonomy support, and moderate nudging, yet also sensitive to the cognitive load demands imposed on learners. Theoretically, combining Persuasive Systems Design and SDT in this work provides a viable framework for explaining the interplay between technology and intrinsic motivation support and challenge. In practice, these findings guide platform builders, teachers, and policymakers on what the optimal design principles are for engaging not just short-term compliance but self-directed learning commitment in the long run.

### 5.2. Mediation Analysis: Uncovering the Psychological Mechanisms Underlying Persuasive Learning Design

In order to enhance dismantling the processes by which persuasive factors on learning platforms affect intrinsic learning motivation, in this research, cognitive overload (COG) and perceived autonomy (PAUTO) were examined as mediators. Mediation analysis yields informative evidence not only about whether psychological processes moderate platform feature effects on learner motivation, but also how and when such effects occur. The findings establish an interactive sophistication between rival and complement mediation forms, respectively, corroborating the necessity of balancing cognitive and motivational demands subtly in persuasive learning environments.

Perceived Persuasiveness of Platform Design (PPS) was a double-edged sword. Directly, the influence of PPS on intrinsic motivation (INTR) was positive and significant, whereas both indirect effects—through cognitive overload (H5a) and loss of autonomy (H5b)—were negative and statistically significant. This phenomenon illustrates competitive partial mediation, where the total motivational effect of persuasive elements is both motivated and demotivated by opposing processes. Conceptually, this tension is reminiscent of experiments in Self-Determination Theory, which assumes that controlling design—under good intentions—erodes users’ felt autonomy and intrinsic motivation (Kian et al., 2022; Schmid & Schoop, 2022). From the Cognitive Load Theory perspective, placing the persuasive cues will also heighten the extraneous load if introduced features pull attention away from central learning material. The consideration in design is that, whereas short-term motivational effects may be triggered by the provision of gamified badges or progress markers, the long-term impact will be contingent upon keeping their psychological cost to a minimum. Interface developers must ensure that such functions are not overburdensome for learners and do not compromise their volitional control (Dicheva et al., 2023; Fan, 2023; Ismailov & Ono, 2021).

Conversely, Nudge Exposure (NE) revealed a complement partial mediation model, where the indirect effects supplemented the existing significant direct relationship to intrinsic motivation. More specifically, NE on reduced cognitive overload (H6a) and enhanced perceived autonomy (H6b) both had positive effects on intrinsic motivation. These findings are consistent with existing research on digital nudging as a model, which, if applied ethically and in conformity with users’ tendencies, may facilitate auto-manifested behavior and self-directed learning (Adaji & Adisa, 2022; Brown et al., 2023; Jin & Bridges, 2014). Instead of being felt as coercive, nudges here—like streak reminders or progress suggestions—can be welcomed by students as helpful aids to decision and verification of progress without the exercise of coercive pressure. This is contrary to the suggestion that nudges always undermine autonomy and, rather, highlights framing, frequency, and control by the user to nudge success (Kovtaniuk et al., 2025; Srivastava et al., 2024).

Perceived Personalization (PP) showed the strongest pattern of mediation in the model. Both indirect routes—via decreased cognitive overload (H7a) and increased autonomy (H7b)—were highly significant and supported a complementary mediation model. Since students considered content to require shaping to their needs, goals, or learning history, students experienced both greater self-determination and reduced cognitive overload, both of which elicited deeper motivational investment. These results support Self-Determination Theory, in which autonomy-supportive environments are seen as the prerequisite to intrinsic motivation, and Expectancy–Value Theory, according to which individualized learning environments enhance perceived relevance and value of the task. Counterintuitively, PP accounted for the greatest-sum indirect effect, such that personalization is not just a surface feature of design, but a deep psychological motivator of engagement. The collective interpretation of such mediation results is as follows: design features that maximize learner autonomy and minimize cognitive load are likely to induce intrinsic motivation (Knight, 2025; Martin & Kwaku, 2019; F. A. Orji et al., 2024). To that end, personalization and ethical nudging are suitable strategies, and persuasive design features need to be applied with care since the danger is that they increase cognitive demands indirectly or lower experienced agency. It is not only theoretically intriguing, but also downright essential in the development of persuasive educational systems where the distinction between motivation and manipulation is narrow and sensitive to context (Appel & Fernández, 2022; Bozkurt et al., 2020).

Our PPS competitive mediation implies building simple reportable indicators of subjective experience. Initially, an Autonomy Index (the PAUTO composite) and an Overload Index (the COG composite) can be monitored alongside engagement. Secondly, a tractable Persuasion–Autonomy Gap can be computed as the gap between PPS’s direct payoff and the sum of the negative indirect effects via PAUTO and COG; the increasing gap indicates motivational gain bought at psychological expense. Having posted such metrics (with frequency, timing, and dismissibility-of-cues guardrails), ethical-by-design commitments would be put into action.

Together, the mediation analysis contributes theoretical insight into how persuasive design, nudging, and personalization engage with learners’ motivational and cognitive states. It validates that not all persuasion is motivating equally, and that design decisions must foreground autonomy and cognitive clarity if they are to foster sustainable self-determined learning. These results provide actionable recommendations to education technology designers, instructors, and policymakers interested in mapping digital platform features to evidence-based theories of learner engagement and motivation (see Table 8).

### 5.3. Multi-Group Analysis: Demographic and Experiential Moderators of Digital Persuasion Dynamics

The findings of the Multi-Group Analysis (MGA) provide provocative evidence on the differential impact of persuasive, personalized, and autonomy-supportive features in online learning platforms across meaningful demographic and experiential subgroups. The evidence suggests that context sensitivity in platform design is important as it is reported that student traits like gender, age, level of education, digital proficiency, and frequency of use of the platform are involved in establishing the motivational effects of technology-based interventions.

Gender served as a significant moderator for a number of model paths. Male subjects were more sensitive to system-level design elements, showing greater effects of perceived persuasiveness (PPS) and cognitive overload (COG) on intrinsic motivation (INTR). These results are consistent with previous research indicating men would be more sensitive to competitive or structure-related cues. Female participants, in contrast, were more susceptible to nudging (NE) and personalization (PP), specifically in their influence on perceived autonomy (PAUTO), resonating with findings on gendered appreciation of affectively supportive and personalized settings (Adaji & Adisa, 2022; Aldenaini et al., 2020; Brown et al., 2023). These differences indicate the potential benefit of gender-sensitive design that incorporates cognitive stimulation with emotional appeal.

Age differences reveal that students’ level of development and metacognitive preparedness impact their reaction to aspects of the platform. Young adults (18–20) were more susceptible to being nudged with regard to autonomy but were more prone to overload due to adaptive aspects. Older adults (27–29) were less prone to overload and had greater autonomy-based motivation, particularly to persuasive design. These trends inform adult learning and cognitive development theories so that education technologies must be responsive to adjust for age difference in self-management and cognitive processing (Chapman et al., 2025; Hocine & Sehaba, 2024).

Educational level also moderated a number of paths. Those with Bachelor’s and Master’s degrees exhibited more robust autonomy responses for personalization and persuasive design and higher internalization of motivation. This can be attributed to earlier evidence that correlated experience of education with greater metacognitive awareness and autonomy preference (Inocencio, 2018; Jin & Bridges, 2014). Lower educated users, on the other hand, experienced greater cognitive overload, emphasizing the need to decrease platform interfaces and remove unnecessary load from novice learners.

Digital literacy had a powerful effect on the motivational value of personalization and the cognitive expense of persuasion. High-literacy users gained more from personalized functions and choice-led paths, whereas low-literacy users had the increased negative effects of overload and nudging, which emphasized the risk of unwished-for cognitive loads any time persuasive mechanisms are not aligned with the technical skill of users. These results satisfy new demands in the persuasive technology literature to expose digital disparity within adaptive system design (Murillo-Muñoz et al., 2021; Ning et al., 2025).

Persuasive element understanding also moderated user experience. Novice users were more prone to the motivational benefits and cognitive costs of persuasive design, while experienced users were more stable and moderated in response, possibly due to being more aware or habituated. This indicates that persuasive systems might need onboarding or adaptive scaffolding to mitigate disorientation in novices.

Lastly, frequency of use played a two-dimensional role. Heavy users reacted positively to nudging but negatively to autonomy, while light users reacted positively to overload and persuasive design. This implies an exposure effect where heavy usage increases responsiveness to behavioral cues but decreases novelty or perceived autonomy of the system. These results indicate that motivational strategies need to be situational, varying based on user tenure and interaction history. MGA patterns suggest that such interventions must be subgroup-sensitive: to low-familiarity/low-digital-literacy users, lower caps and stricter limits on nudge frequencies are necessary; to high-frequency users, higher limits are acceptable if autonomy is high. In practice, sites can vary nudge dose and chatbot proactivity on age, literacy, and familiarity cues and, in real time, on autonomy and overload measures.

Overall, the MGA findings underscore the value of adaptive and persuasive learning systems to bridge an overly reductive one-size-fits-all remedy. Learners’ demographic, education, and digital environments should be considered by designers and educators to implement adaptive strategies that promote autonomy, minimize overload, and accommodate intrinsic motivation. Multistep adaptation processes that modulate persuasion strength, message frames, and interface complexity over time to maximize learning outcomes for various user groups are a research domain in need of further work.

## 6. Practical Implications

The results of the present study offer useful insights for stakeholders in education technology platform development, adoption, and regulation. As the learning environment is increasingly filled with adaptive and persuasive technologies ranging from gamification elements and nudges to personalization algorithms, the cognitive and motivational impacts of such designs on learning must be tested comprehensively. The findings here indicate the potential and limits of persuasive systems for learning and posit that more insidious user-sensitive approaches are required for maximizing engagement and protecting learner well-being.

### 6.1. Implications for Educational Technology Designers and Platform Developers

To EdTech designers and UX designers, the findings imply a double imperative: to maximize the motivational potential of persuasion factors at as low an unintended cognitive or psychological cost as possible per benefit. The competitive mediation pattern between perceived persuasiveness and intrinsic motivation—persuasive design increases motivation directly but also provokes cognitive overload and diminishes autonomy—implies the value of constraint in persuasive user interface design. Badges, prompts, and gamified feedback are all methods that need to be applied wisely and in sync with students’ developmental and cognitive stages (Plak et al., 2023; Shi et al., 2025; Wenker, 2022).

Moreover, the powerful positive impact of Perceived Personalization on autonomy and intrinsic motivation attests to the benefits of adaptive learning sequences, customized content suggestions, and adaptive feedback mechanisms. However, designers need to beware that the features will not overwhelm users or lead to interface complexity, especially for less digitally literate or less educationally adept users. This requires informed personalization systems that balance adaptivity and simplicity and provide opt-in customization instead of forced adaptation.

Operationally, EdTech systems need to have user modeling engines in place to monitor digital fluency, previous experience, and pattern of use and to modify the force or character of persuasive interventions (Plak et al., 2023; Shi et al., 2025; Wenker, 2022). Onboarding guidance, autonomy-supporting feedback, and explanation of persuasive aims need to be integrated in systems so that they are able to establish confidence and avoid manipulation apprehension.

### 6.2. Implications for Educators and Instructional Designers

Educators are at the forefront of unpacking and negotiating the implications of persuasion technologies in teaching and learning. This study demonstrates that teachers need to be ready to recognize how features of online platforms map onto students’ cognition and motivation, particularly in blended learning or fully online learning environments.

That both promote intrinsic motivation and perceived autonomy—when supportively framed—implies that instructional design can also benefit from the same principles. That is, reminders, goal-setting prompts, or positive reinforcement messages of encouragement, when strategically placed, can be utilized to help hold learner attention without destroying autonomy (Yin et al., 2021; Zhang, 2023). Instructors must also be sensitive to signs of overload or disengagement, especially for learners who are new to persuasive technologies or low in digital literacy.

Instructional designers are advised to incorporate the application of scaffolding mechanisms and reflection points within the curriculum so that students can critically reflect on nudges and personalization cues. In addition, differential motivational responses in terms of gender and age imply the requirement for instruction with equally customized instructional strategies (Shi et al., 2025; Wenker, 2022). Female students can be more effectively instructed using emotionally calming messages and adaptive pacing, whereas male students can be more effectively motivated using achievement-related factors and system-level persuasion. LLM-driven coaches with (a) transparency markers (why this request, how calm), (b) user controls (snooze, mute, opt-out), (c) frequency limits and quiet time, (d) rationales explaining in terms of learning goals, and (e) audit logs for tracking instruction can be implemented. Autonomy and overload highest-ranked product KPIs can be prioritized, and chatbot initiatives can be adjusted to reduce the Persuasion–Autonomy Gap for subgroups.

### 6.3. Implications for Educational Policymakers and Administrators

At the system level, education policymakers need to identify the dual nature of persuasive digital design. Though these technologies provide mass-produced means of motivation boost and self-learning, they create psychological pressure, manipulation risks, and skewed learning opportunities. The Multi-Group Analysis of the study indicated how the same persuasive elements can have disproportionate impacts across different subgroups of users, widening inequalities if not well tuned (Brown et al., 2023; Chapman et al., 2025; Plak et al., 2023).

Policymakers should, thus, call for the creation of ethical design standards for influential learning technologies as part of a broader innovation in “humane technology” digital governance. The standards would make it the norm to be transparent, include opt-out provision for some nudges, and be required to test cognitive load prior to deployment. In addition, school districts and college administrations will have to turn to equity-based purchasing standards in choosing computer-based learning products. Systems that are shown to be inclusive within demographic categories—personalization without overloading, autonomy without oppression, and engaging functionality that can accommodate multiple user profiles—have to be given priority.

Digital literacy programs should also be integrated throughout education levels. The consequences that vulnerability to persuasion and mental overload are more common among low-literacy consumers imply that acquisition of digital skills is a requirement for equal access to prevailing learning environments. In-service training opportunities for teachers and students can enhance critical awareness of persuasive design so that users may perform more independently and efficiently with EdTech devices.

### 6.4. Implications for EdTech Business Managers and Innovation Leaders

For EdTech business decision makers, guidance is offered on applying persuasive techniques not only to optimize engagement metrics, but to optimize long-term learner satisfaction and retention. Too-heavy-handed nudging or obtrusively personalized individualized experiences will win short-term click-through or time-on-platform but lose in the long term by inducing dropout or ill will (Chapman et al., 2025; Hocine & Sehaba, 2024; Murillo-Muñoz et al., 2021). The competitive and complementary mediation patterns found in this study highlight the necessity for a change from short-term behavioral manipulation to long-term motivational sustainability. Business as usual in business models must look to ethical engagement and not just define success in behaviorist outcomes, but also outcomes of learner autonomy, satisfaction, and development. Product teams would be motivated to implement adaptive persuasion structures that would be fine-tuned using real-time feedback and subgroup response, as well as A/B testing suites that incorporate psychological outcomes into their measurement metric. Innovation leaders who adopt more holistic design thinking can make platforms that are principled and persuasive, achieving maximum effect without hurting user trust (Shi et al., 2025; Wenker, 2022).

## 7. Conclusions, Limitations, and Future Research Directions

This research aimed to replicate the influence of persuasive design, frequency nudging, and Perceived Personalization on intrinsic motivation in online learning environments with mediation by cognitive overload (COG) and perceived autonomy (PAUTO). Structural Equation Modeling (SEM) verified that all three platform features have a significant direct and indirect influence on motivation. Complementary mediation was found for nudging and personalization—crafting autonomy and overload reduction—while persuasive design uncovered competitive mediation, boosting motivation but simultaneously raising cognitive tension and lowering autonomy. Additional insights were drawn from Multi-Group Analysis (MGA), which unveiled the ways that user traits, i.e., gender, age, education, digital literacy, level of familiarity with persuasive functions, and frequency of platform use, impact the mechanisms differently.

This research is not without limitations. To further establish the worth of such findings, future research would be assisted by utilizing a longitudinal or experimental design in an effort to make stronger inferences regarding causality and temporal processes (Fan, 2023; Ismailov & Ono, 2021; Schmid & Schoop, 2022). Although this study provides us with a high-resolution image of psychological process in persuasive EdTech, longitudinal development might show the extent to which learners gradually accommodate repeated exposure to adaptive and persuasive elements, e.g., whether motivational gains from early periods are sustained, eroded, or reconfigured over time. A useful approach would be to triangulate self-reported psychological data with behavioral and physiological measures. Human-centered modeling in the current model depended on psychometrically well-validated measures, but aggregation of measures like eye movements, clickstream data, or task completion would yield denser moment-to-moment information regarding the process of cognitive load and autonomy in interaction (Suriagiri et al., 2022; Terkaj et al., 2024; Yu et al., 2023). Multi-modal evidence for this would be capable of separating perceived and actual cognitive burden, a useful step in the process of optimizing system design.

Moreover, this research was aimed at a specific sample of university students with a specific learning context. Future research would aim at heterogeneous user groups and learning contexts, i.e., K–12 students, professional adult workers in vocational training, or corporate workers in business learning systems. Such a comparative design would establish whether, and to what extent, mechanisms generalize across different developmental, cultural, or organizational contexts, and whether support strategies should be adapted to the context (Dicheva et al., 2023; Fan, 2023; Ismailov & Ono, 2021). An exciting avenue for future work is the real-time adaptive response of persuasive systems to user profiles. This study’s MGA findings emphasize that persuasion, nudges, and personalization do not work for all, but interact with personality. Future systems can, thus, leverage AI-driven personalization that modulates the level and nature of persuasive functionality in response to demographic, behavioral, or psychological cues to maximize motivation without inducing strain. Likewise, the current model concentrated on cognition and motivation mediators but other processes including emotional involvement, perceived fairness, or user trust can impact the line from design to motivation as well. Incorporation of such constructs into models of the future can potentially deepen understanding of how rich digital contexts affect learner psychology beyond cognition and autonomy only (Hocine & Sehaba, 2024; Inocencio, 2018; Jin & Bridges, 2014). Lastly, future research needs to consider the ethical obligations of persuasive and adaptive functionalities, particularly on vulnerable populations. The distinction between motivation and manipulation continues to be thin, and system transparency, user consent, and online literacy need to be moved front and center in both research and design. Future research can investigate how sensitivity to ethics and perceived transparency intervene in the adoption and efficacy of persuasive technologies (Dicheva et al., 2023; Fan, 2023; Ismailov & Ono, 2021).

Combined, these findings represent a subtle dance among design, cognition, and human motivation in online learning. Persuasive design practices are neither good nor bad on their own, but as tools whose effect hinges on their engaging with the learner’s inner life, their bandwidth, their need for autonomy, and their willingness to participate. Like a good symphony, the greatest learning systems digitally do not merely teach or persuade; they inspire. They predict resistance without coercion, grant guidance without intrusiveness, and enhance development without detracting from self-regulation. As we move towards the future, the task of design and research is not simply to maximize rates of engagement, but to design spaces of learning that engage mind and spirit alike, spaces that invoke behavior but foster possibility. In such a system, learning is not an accomplishment, but an embracing.

## Figures and Tables

**Table 1:** Sample profile.

		Frequency	Percentage
**Gender**	Female	374	50.5%
	Male	366	49.5%
**Age**	18–20	109	14.7%
	21–23	192	25.9%
	24–26	161	21.8%
	27–29	164	22.2%
	30+	114	15.4%
**Study Level**	High school diploma	109	14.7%
	Undergraduate student	245	33.1%
	Bachelor’s degree	209	28.2%
	Master’s degree or higher	177	23.9%
**How often do you use smart learning platforms for your studies?**	Rarely/only when required	184	24.9%
	A few times per month	160	21.6%
	Once a week	114	15.4%
	Several times a week	129	17.4%
	Daily	153	20.7%
**How familiar are you with features like badges, reminders, streaks, or rewards in learning platforms?**	Not familiar at all—I do not notice such features	219	29.6%
	Not very familiar—I rarely notice or use them	138	18.6%
	Somewhat familiar—I occasionally use them	176	23.8%
	Very familiar—I notice and use them frequently	207	28.0%
**What device do you most often use to access learning platforms?**	Smartphone	156	21.1%
	Laptop	158	21.4%
	Desktop computer	178	24.1%
	Tablet	86	11.6%
	Other	162	21.9%
**How would you rate your overall digital literacy? (your confidence in using online platforms and tools)**	Very low	123	16.6%
	Low	90	12.2%
	Moderate	46	6.2%
	High	129	17.4%
	Very high	352	47.6%
**Which of the following smart learning platforms have you used in the past 3 months?**	Moodle	134	18.1%
	Coursera	111	15.0%
	Duolingo	51	6.9%
	Khan Academy	140	18.9%
	YouTube (for learning)	169	22.8%
	Other	135	18.2%

**Table 2:** Factor loading reliability and convergent validity.

Constructs	Items	Factor Loadings	Cronbach’s Alpha	rho_A	CR	AVE
Cognitive Overload	COG1	0.723	0.652	0.697	0.807	0.584
	COG2	0.847				
	COG3	0.716				
Intrinsic Motivation for Learning	INTR1	0.773	0.832	0.839	0.901	0.752
	INTR2	0.906				
	INTR3	0.915				
Frequency of Nudge Exposure	NE1	0.754	0.793	0.803	0.865	0.617
	NE2	0.731				
	NE3	0.838				
	NE4	0.814				
Perceived Autonomy	PAUTO1	0.889	0.882	0.887	0.927	0.809
	PAUTO2	0.913				
	PAUTO3	0.896				
Perceived Personalization	PP1	0.888	0.918	0.919	0.942	0.804
	PP2	0.859				
	PP3	0.921				
	PP4	0.916				
Perceived Persuasiveness of Platform Design	PPS1	0.772	0.832	0.919	0.876	0.587
	PPS2	0.884				
	PPS3	0.799				
	PPS4	0.628				
	PPS5	0.726				

**Table 3:** HTMT ratio.

	COG	INTR	NE	PAUTO	PP	PPS
COG						
INTR	0.619					
NE	0.697	0.661				
PAUTO	0.619	0.654	0.529			
PP	0.818	0.717	0.692	0.729		
PPS	0.298	0.126	0.177	0.247	0.130	

**Note:** This table contains HTMT ratios between all pairs of latent constructs. Below 0.85 is the cut-off for adequate discriminant validity. All such estimates in this analysis are above that, providing evidence that each construct is empirically separate.

**Table 4:** Fornell and Larcker criterion.

	COG	INTR	NE	PAUTO	PP	PPS
COG	**0.764**					
INTR	0.486	**0.867**				
NE	0.526	0.542	**0.786**			
PAUTO	0.471	0.560	0.457	**0.899**		
PP	0.651	0.625	0.598	0.658	**0.896**	
PPS	−0.203	−0.002	−0.045	−0.235	−0.105	**0.766**

**Note:** The diagonal values (in bold) represent the square roots of the AVE for each construct, which should be greater than the inter-construct correlations in the corresponding rows and columns. This condition is met across all constructs, supporting discriminant validity in the measurement model.

**Table 5:** Hypotheses testing.

Hypothesis	Path	Coefficient (β)	SD	t-Value	*p*-Value	Results
H1	PPS → INTR	0.118	0.033	3.606	0.000	Supported
H2	NE → INTR	0.211	0.034	6.250	0.000	Supported
H3	PP → INTR	0.273	0.044	6.268	0.000	Supported
H4a	COG → INTR	0.096	0.039	2.474	0.007	Supported
H4b	PAUTO → INTR	0.267	0.038	6.948	0.000	Supported

**Table 6:** Mediation analysis.

Hypothesis	Direct Effects	Coeff. (β)	SD	t-Value	*p*-Value	Results	Mediation Type
	PPS → INTR	0.118	0.033	3.606	0.000		
	NE → INTR	0.211	0.034	6.250	0.000		
	PP → INTR	0.273	0.044	6.268	0.000		
	**Total Effects**	**Coeff. (β)**	**SD**	**t-value**	***p*-value**		
	NE → INTR	0.049	0.015	3.220	0.001		
	PP → INTR	0.203	0.029	6.944	0.000		
	PPS → INTR	−0.059	0.013	4.686	0.000		
	**Specific Indirect Effects**	**Coeff. (β)**	**SD**	**t-value**	***p*-value**		
H5a	PPS → COG → INTR	−0.013	0.005	2.464	0.007	Supp.	Competitive Partial Med.
H5b	PPS → PAUTO → INTR	−0.045	0.011	4.137	0.000	Supp.	Competitive Partial Med.
H6a	NE → COG → INTR	0.021	0.010	2.155	0.016	Supp.	Complementary Partial Med.
H6b	NE → PAUTO → INTR	0.028	0.011	2.555	0.005	Supp.	Complementary Partial Med.
H7a	PP → COG → INTR	0.048	0.019	2.485	0.006	Supp.	Complementary Partial Med.
H7b	PP → PAUTO → INTR	0.154	0.022	6.917	0.000	Supp.	Complementary Partial Med.

**Table 7:** Significant MGA paths with group comparisons.

Path	Group Comparison	Difference (Δβ)	*p*-Value
COG → INTR	Male vs. Female	0.427	0.000
NE → INTR	Male vs. Female	−0.274	0.000
PP → INTR	Male vs. Female	−0.306	0.000
PPS → INTR	Male vs. Female	0.437	0.000
PPS → COG	Male vs. Female	0.200	0.006
NE → PAUTO	Male vs. Female	−0.181	0.008
NE → PAUTO	18–20 vs. 21–23	0.510	0.000
NE → PAUTO	18–20 vs. 24–26	0.243	0.045
NE → PAUTO	18–20 vs. 27–29	0.304	0.020
NE → PAUTO	21–23 vs. 24–26	−0.267	0.004
NE → PAUTO	21–23 vs. 27–29	−0.205	0.027
PP → INTR	18–20 vs. 21–23	−0.640	0.000
PP → INTR	21–23 vs. 24–26	0.847	0.000
PP → INTR	21–23 vs. 27–29	0.612	0.000
PP → PAUTO	18–20 vs. 21–23	−0.469	0.000
PP → PAUTO	18–20 vs. 24–26	−0.300	0.012
PP → PAUTO	18–20 vs. 27–29	−0.326	0.008
PP → PAUTO	21–23 vs. 24–26	0.168	0.044
NE → COG	18–20 vs. 21–23	−0.410	0.002
NE → COG	21–23 vs. 24–26	0.363	0.000
NE → COG	21–23 vs. 27–29	0.316	0.000
PP → COG	18–20 vs. 21–23	0.312	0.013
PP → COG	21–23 vs. 24–26	−0.217	0.026
PP → COG	21–23 vs. 27–29	−0.293	0.001
NE → INTR	18–20 vs. 21–23	0.244	0.015
NE → INTR	18–20 vs. 27–29	0.247	0.022
PPS → COG	18–20 vs. 21–23	0.234	0.025
PPS → COG	18–20 vs. 24–26	0.211	0.034
PPS → COG	21–23 vs. 27–29	−0.411	0.001
PPS → COG	24–26 vs. 27–29	−0.388	0.004
COG → INTR	18–20 vs. 21–23	0.268	0.029
COG → INTR	21–23 vs. 24–26	−0.309	0.038
PPS → INTR	21–23 vs. 27–29	−0.356	0.026
PAUTO → INTR	18–20 vs. 27–29	−0.242	0.012
PAUTO → INTR	21–23 vs. 27–29	−0.299	0.003
PPS → PAUTO	18–20 vs. 24–26	0.273	0.006
PPS → PAUTO	21–23 vs. 24–26	0.273	0.002
PP → PAUTO	Bachelor’s—High School	0.294	0.002
PP → PAUTO	High School—Undergraduate	−0.185	0.035
PPS → PAUTO	Bachelor’s—High School	0.302	0.005
PPS → PAUTO	Master’s—Undergraduate	−0.499	0.005
PP → COG	Bachelor’s—High School	0.218	0.014
NE → COG	Bachelor’s—High School	−0.162	0.044
PPS → INTR	Master’s—High School	0.256	0.029
PAUTO → INTR	Master’s—High School	0.268	0.007
PAUTO → INTR	Master’s—Undergraduate	0.211	0.017
PPS → COG	Master’s—Undergraduate	−0.337	0.017
PPS → COG	High School—Undergraduate	−0.241	0.032
COG → INTR	Bachelor’s—Master’s	−0.335	0.004
COG → INTR	Bachelor’s—Undergraduate	−0.258	0.011
NE → INTR	Bachelor’s—Undergraduate	−0.199	0.021
NE → INTR	Master’s—Undergraduate	−0.237	0.005
NE → PAUTO	Master’s—Undergraduate	−0.222	0.029
COG → INTR	Digital Literacy—High vs. Low	−0.253	0.000
PP → INTR	Digital Literacy—High vs. Low	0.469	0.000
PP → PAUTO	Digital Literacy—High vs. Low	0.267	0.000
NE → PAUTO	Digital Literacy—High vs. Low	−0.250	0.001
PAUTO → INTR	Digital Literacy—High vs. Low	−0.207	0.003
NE → INTR	Digital Literacy—High vs. Low	−0.131	0.022
PP → COG	Digital Literacy—High vs. Low	0.137	0.037
COG → INTR	Familiarity—High vs. Low	−0.310	0.000
NE → PAUTO	Familiarity—High vs. Low	0.291	0.000
PAUTO → INTR	Familiarity—High vs. Low	−0.285	0.000
PP → PAUTO	Familiarity—High vs. Low	−0.228	0.000
NE → INTR	Familiarity—High vs. Low	0.236	0.001
PPS → INTR	Familiarity—High vs. Low	−0.261	0.001
PP → INTR	Familiarity—High vs. Low	0.166	0.037
PPS → COG	Familiarity—High vs. Low	−0.120	0.039
NE → INTR	High vs. Low Frequency of Use	0.390	0.000
PAUTO → INTR	High vs. Low Frequency of Use	−0.330	0.000
PPS → INTR	High vs. Low Frequency of Use	−0.184	0.005
COG → INTR	High vs. Low Frequency of Use	−0.194	0.006
PP → COG	High vs. Low Frequency of Use	−0.135	0.025
PPS → COG	High vs. Low Frequency of Use	−0.135	0.038
PPS → PAUTO	High vs. Low Frequency of Use	0.106	0.052

**Table 8:** Summary of key findings.

Hypothesis	Path	Mediation Type	Interpretation
H1	PPS → INTR	—	Direct positive influence of persuasive design on motivation
H2	NE → INTR	—	Direct positive influence of nudge frequency on motivation
H3	PP → INTR	—	Direct positive influence of personalization on motivation
H5a	PPS → COG → INTR	Competitive Partial Med.	Cognitive overload diminishes the beneficial impact of PPS
H5b	PPS → PAUTO → INTR	Competitive Partial Med.	Adverse indirect effects stifle direct advantages of PPS
H6a	NE → COG → INTR	Complementary Partial Med.	Nudges minimize overload, which consolidates motivation
H6b	NE → PAUTO → INTR	Complementary Partial Med.	Indirect effects strengthen direct motivational impacts of nudging
H7a	PP → COG → INTR	Complementary Partial Med.	Personalization alleviates mental effort
H7b	PP → PAUTO → INTR	Complementary Partial Mediation	Personalized elements support autonomy and motivation

## Data Availability

The data presented in this study are available on request from the corresponding author.

## Appendix A

**Table A1:** Measurements used for data analysis.

**Perceived Persuasiveness of Platform Design (PPS)**		
PPS1	This platform’s features are designed to influence my behavior.	Adapted from (Thomas et al., 2019)
PPS2	I feel that badges, reminders, and progress bars push me to act in a certain way.	
PPS3	The system seems to try to persuade me rather than inform me.	
PPS4	The platform encourages me to continue even when I do not plan to.	
PPS5	The design feels more like digital marketing than education.	
**Frequency of Nudge Exposure (NE)**		
NE1	I frequently receive reminders or pop-ups encouraging me to continue learning.	Adapted from (Brick et al., 2023)
NE2	I feel nudged by design features more than once during each session.	
NE3	I often see badges or streaks based on my activity.	
NE4	The platform regularly prompts me to complete extra tasks.	
NE5	I notice progress bars or motivational messages almost every time I log in. (deleted)	
**Perceived Personalization (PP)**		
PP1	The content I see on the platform feels personally relevant to me.	Adapted from (Aguirre et al., 2015)
PP2	I believe this platform understands my learning needs.	
PP3	The lessons or suggestions are tailored to my progress.	
PP4	I recognize my own situation in the way the material is presented.	
PP5	The feedback I receive feels customized. (deleted)	
**Cognitive Overload (COG)**		
COG1	I feel mentally overloaded when using this platform.	Adapted from (Choi & Lee, 2022)
COG2	I feel pressured to keep up with the platform’s prompts.	
COG3	I feel anxious when I do not meet the system’s expectations.	
**Perceived Autonomy (PAUTO)**		
PAUTO1	I feel that I have a lot of control over how I use this platform.	Adapted from (Bosch, 2024)
PAUTO2	I can make my own choices while learning here.	
PAUTO3	My learning path feels self-directed, not imposed.	
**Intrinsic Motivation for Learning (INTR)**		
INTR1	I use this platform because I want to, not because I have to.	Adapted from (Bosch, 2024)
INTR2	Learning with this system is fun and rewarding.	
INTR3	I feel motivated to continue learning through this platform.	

## References

[B1-ejihpe-15-00179] Adaji I., Adisa M. (2022). A review of the use of persuasive technologies to influence sustainable behaviour. 30th ACM Conference on User Modeling, Adaptation and Personalization.

[B2-ejihpe-15-00179] Aguirre E., Mahr D., Grewal D., De Ruyter K., Wetzels M. (2015). Unraveling the personalization paradox: The effect of information collection and trust-building strategies on online advertisement effectiveness. Journal of Retailing.

[B3-ejihpe-15-00179] Akgün Ö. E., Topal M. (2018). The Turkish adaptation study of the gamification user types hexad scale. International Journal of Assessment Tools in Education.

[B4-ejihpe-15-00179] Aldenaini N., Alqahtani F., Orji R., Sampalli S. (2020). Trends in persuasive technologies for physical activity and sedentary behavior: A systematic review. Frontiers in Artificial Intelligence.

[B5-ejihpe-15-00179] Alslaity A., Chan G., Orji R. (2023). A panoramic view of personalization based on individual differences in persuasive and behavior change interventions. Frontiers in Artificial Intelligence.

[B6-ejihpe-15-00179] Antoniadou M., Rahiotis C., Kakaboura A. (2022). Sustainable distance online educational process for dental students during COVID-19 pandemic. International Journal of Environmental Research and Public Health.

[B7-ejihpe-15-00179] Appel C., Fernández S. S., Auer M. E., Pester A., May D. (2022). Reimagining language learning in higher education: Key-roles for technology. Learning with technologies and technologies in learning.

[B8-ejihpe-15-00179] Asad M. M., Erum D., Churi P., Moreno Guerrero A. J. (2023). Effect of technostress on psychological well-being of post-graduate students: A perspective and correlational study of higher education management. International Journal of Information Management Data Insights.

[B9-ejihpe-15-00179] Bosch C. (2024). Assessing the psychometric properties of the intrinsic motivation inventory in blended learning environments. Journal of Education and E-Learning Research.

[B10-ejihpe-15-00179] Bozkurt A., Jung I., Xiao J., Vladimirschi V., Schuwer R., Egorov G., Lambert S. R., Al-Freih M., Pete J., Olcott D., Rodes V., Aranciaga I., Alvarez A. V., Roberts J., Pazurek A., Raffaghelli J. E. (2020). A global outlook to the interruption of education due to COVID-19 Pandemic: Navigating in a time of uncertainty and crisis. Asian Journal of Distance Education.

[B11-ejihpe-15-00179] Brick K., De Martino S., Visser M. (2023). Behavioural nudges for water conservation in unequal settings: Experimental evidence from Cape Town. Journal of Environmental Economics and Management.

[B12-ejihpe-15-00179] Brown A., Basson M., Axelsen M., Redmond P., Lawrence J. (2023). Empirical evidence to support a nudge intervention for increasing online engagement in higher education. Education Sciences.

[B13-ejihpe-15-00179] Chapman J. R., Kohler T. B., Rich P. J., Trego A. (2025). Maybe we’ve got it wrong. An experimental evaluation of self-determination and flow theory in gamification. Journal of Research on Technology in Education.

[B14-ejihpe-15-00179] Cheah J.-H., Amaro S., Roldán J. L. (2023). Multigroup analysis of more than two groups in PLS-SEM: A review, illustration, and recommendations. Journal of Business Research.

[B15-ejihpe-15-00179] Cheng X., Bao Y., Yang B., Chen S., Zuo Y., Siponen M. (2023). Investigating students’ satisfaction with online collaborative learning during the COVID-19 period: An expectation-confirmation model. Group Decision and Negotiation.

[B16-ejihpe-15-00179] Cheung S. L., Rogut N. (2024). Portfolio framing and diversification in a disposition effect experiment. Journal of Behavioral and Experimental Finance.

[B17-ejihpe-15-00179] Chin W. W. (1998). The partial least squares approach to structural equation modeling. Modern Methods for Business Research.

[B18-ejihpe-15-00179] Choi Y., Lee H. (2022). Psychometric properties for multidimensional cognitive load scale in an E-learning environment. International Journal of Environmental Research and Public Health.

[B19-ejihpe-15-00179] Coelho F., Rando B., Aparício D., Pontífice-Sousa P., Gonçalves D., Abreu A. M. (2025). The impact of educational gamification on cognition, emotions, and motivation: A randomized controlled trial. Journal of Computers in Education.

[B20-ejihpe-15-00179] Daniel K., Msambwa M. M., Antony F., Wan X. (2024). Motivate students for better academic achievement: A systematic review of blended innovative teaching and its impact on learning. Computer Applications in Engineering Education.

[B21-ejihpe-15-00179] Das H., Goulas S., Monachou F. (2025). Why students reject AI for human counselors in college applications: A field experiment.

[B22-ejihpe-15-00179] De Ridder D., Kroese F., Van Gestel L. (2022). Nudgeability: Mapping conditions of susceptibility to nudge influence. Perspectives on Psychological Science.

[B23-ejihpe-15-00179] Dicheva D., Guy B., Dichev C., Irwin K., Cassel L. (2023). A multi-case empirical study on the impact of virtual currency on student engagement and motivation. Trends in Higher Education.

[B24-ejihpe-15-00179] Fan C. (2023). English learning motivation with TAM: Undergraduates’ behavioral intention to use Chinese indigenous social media platforms for English learning. Cogent Social Sciences.

[B25-ejihpe-15-00179] Fornell C., Larcker D. F. (1981). Evaluating structural equation models with unobservable variables and measurement error. Journal of Marketing Research.

[B26-ejihpe-15-00179] Gefen D., Straub D. (2005). A practical guide to factorial validity using PLS-Graph: Tutorial and annotated example. Communications of the Association for Information Systems.

[B27-ejihpe-15-00179] Gould D. J., Sawarynski K., Mohiyeddini C. (2022). Academic management in uncertain times: Shifting and expanding the focus of cognitive load theory during COVID-19 Pandemic Education. Frontiers in Psychology.

[B28-ejihpe-15-00179] Hair J. F., Black W. C., Babin B. J., Anderson R. E., Tatham R. (2006). Multivariate data analysis.

[B29-ejihpe-15-00179] Hair J. F., Hult G. T. M., Ringle C. M., Sarstedt M., Danks N. P., Ray S. (2021a). An introduction to structural equation modeling. Partial least squares structural equation modeling (PLS-SEM) using R.

[B30-ejihpe-15-00179] Hair J. F., Hult G. T. M., Ringle C. M., Sarstedt M., Danks N. P., Ray S. (2021b). Partial least squares structural equation modeling (PLS-SEM) using R: A workbook.

[B31-ejihpe-15-00179] Hair J. F., Ringle C. M., Sarstedt M. (2011). PLS-SEM: Indeed a silver bullet. Journal of Marketing Theory and Practice.

[B32-ejihpe-15-00179] Hair J. F., Sarstedt M., Hopkins L., Kuppelwieser V. G. (2014). Partial least squares structural equation modeling (PLS-SEM): An emerging tool in business research. European Business Review.

[B33-ejihpe-15-00179] Henseler J., Ringle C. M., Sarstedt M. (2015). A new criterion for assessing discriminant validity in variance-based structural equation modeling. Journal of the Academy of Marketing Science.

[B34-ejihpe-15-00179] Hocine N., Sehaba K. (2024). A systematic review of online personalized systems for the autonomous learning of people with cognitive disabilities. Human–Computer Interaction.

[B35-ejihpe-15-00179] Huang M., Mohamad Saleh M. S., Zolkepli I. A., Wang L., Leal Filho W., Salvia A. L., Portela De Vasconcelos C. R. (2024). The mediating effect of perceived persuasiveness on the relationship between gamified reward in ant forest and user’s sustainable behaviour in China. An agenda for sustainable development research.

[B36-ejihpe-15-00179] Inocencio F. (2018). Using gamification in education: A systematic literature review. International Conference on Information Systems 2018 (ICIS 2018).

[B37-ejihpe-15-00179] Ismailov M., Ono Y. (2021). Assignment design and its effects on Japanese college freshmen’s motivation in L2 emergency online courses: A qualitative study. The Asia-Pacific Education Researcher.

[B38-ejihpe-15-00179] Jin J., Bridges S. M. (2014). Educational technologies in problem-based learning in health sciences education: A systematic review. Journal of Medical Internet Research.

[B39-ejihpe-15-00179] Kalvit A., Singhvi D. (2025). Online learning for repeated nudging.

[B40-ejihpe-15-00179] Kawa C., Ianiro-Dahm P. M., Nijhuis J. F. H., Gijselaers W. H. (2023). Effects of a nudging cue targeting food choice in a University Cafeteria: A field study. Healthcare.

[B41-ejihpe-15-00179] Kesmodel U. S. (2018). Cross-sectional studies—What are they good for?. Acta Obstetricia et Gynecologica Scandinavica.

[B42-ejihpe-15-00179] Kian T. W., Sunar M. S., Su G. E. (2022). The analysis of intrinsic game elements for undergraduates gamified platform based on learner type. IEEE Access.

[B43-ejihpe-15-00179] Knight S. (2025). Identifying and designing evidence-informed practice, in practice: The case for Pragmatic Evidence Synthesis Matrices (PESM). International Journal of Qualitative Methods.

[B44-ejihpe-15-00179] Knutas A., Van Roy R., Hynninen T., Granato M., Kasurinen J., Ikonen J. (2019). A process for designing algorithm-based personalized gamification. Multimedia Tools and Applications.

[B45-ejihpe-15-00179] Kovtaniuk I. I., Tarasova O. Y., Zakarliuka I. S. (2025). Capabilities of the Canva web service for creating educational videos to improve the effectiveness of the educational process in flipped learning. CTE Workshop Proceedings.

[B46-ejihpe-15-00179] Lam L. W. (2012). Impact of competitiveness on salespeople’s commitment and performance. Journal of Business Research.

[B47-ejihpe-15-00179] Lee H. Y., Park S., Kim E. H., Seo J., Lim H., Lee J. (2024). Investigating the effects of real-time student monitoring interface on instructors’ monitoring practices in online teaching. CHI Conference on Human Factors in Computing Systems.

[B48-ejihpe-15-00179] Martin B., Kwaku Y.-A., Pappas I. O., Mikalef P., Dwivedi Y. K., Jaccheri L., Krogstie J., Mäntymäki M. (2019). Designing at the intersection of gamification and persuasive technology to incentivize energy-saving. Digital transformation for a sustainable society in the 21st Century.

[B49-ejihpe-15-00179] Matthews L. (2017). Applying multigroup analysis in PLS-SEM: A step-by-step process. Partial least squares path modeling: Basic concepts, methodological issues and applications.

[B50-ejihpe-15-00179] McCarthy S., Ertiö T., Fitzgerald C., Kahma N. (2024). Digital sustainability for energy-efficient behaviours: A user representation and touchpoint model. Information Systems Frontiers.

[B51-ejihpe-15-00179] Memon M. A., Ting H., Cheah J.-H., Thurasamy R., Chuah F., Cham T. H. (2020). Sample size for survey research: Review and recommendations. Journal of Applied Structural Equation Modeling.

[B52-ejihpe-15-00179] Murillo-Muñoz F., Navarro-Cota C., Juárez-Ramírez R., Jiménez S., Nieto Hipólito J. I., Molina A. I., Vazquez-Briseno M. (2021). Characteristics of a persuasive educational system: A systematic literature review. Applied Sciences.

[B53-ejihpe-15-00179] Ng D. T. K., Tan C. W., Leung J. K. L. (2024). Empowering student self-regulated learning and science education through ChatGPT: A pioneering pilot study. British Journal of Educational Technology.

[B54-ejihpe-15-00179] Ning J., Ma Z., Yao J., Wang Q., Zhang B. (2025). Personalized learning supported by learning analytics: A systematic review of functions, pathways, and educational outcomes. Interactive Learning Environments.

[B55-ejihpe-15-00179] Nitzl C., Roldan J. L., Cepeda G. (2016). Mediation analysis in partial least squares path modeling: Helping researchers discuss more sophisticated models. Industrial Management & Data Systems.

[B56-ejihpe-15-00179] Olsen C., St George D. M. M. (2004). Cross-sectional study design and data analysis. College Entrance Examination Board.

[B57-ejihpe-15-00179] Orji F. A., Gutierrez F. J., Vassileva J., Baghaei N., Ali R., Win K., Oyibo K. (2024). Exploring the influence of persuasive strategies on student motivation: Self-determination theory perspective. Persuasive technology.

[B58-ejihpe-15-00179] Orji R., Tondello G. F., Nacke L. E. (2018). Personalizing persuasive strategies in gameful systems to gamification user types. 2018 CHI Conference on Human Factors in Computing Systems.

[B59-ejihpe-15-00179] Pervan M., Curak M., Pavic Kramaric T. (2017). The influence of industry characteristics and dynamic capabilities on firms’ profitability. International Journal of Financial Studies.

[B60-ejihpe-15-00179] Plak S., Van Klaveren C., Cornelisz I. (2023). Raising student engagement using digital nudges tailored to students’ motivation and perceived ability levels. British Journal of Educational Technology.

[B61-ejihpe-15-00179] Podsakoff P. M., MacKenzie S. B., Lee J.-Y., Podsakoff N. P. (2003). Common method biases in behavioral research: A critical review of the literature and recommended remedies. Journal of Applied Psychology.

[B62-ejihpe-15-00179] Podsakoff P. M., MacKenzie S. B., Podsakoff N. P. (2012). Sources of method bias in social science research and recommendations on how to control it. Annual Review of Psychology.

[B63-ejihpe-15-00179] Preacher K. J., Hayes A. F., Hayes A. F., Slater M. D., Snyder L. B. (2008). Contemporary approaches to assessing mediation in communication research. The Sage sourcebook of advanced data analysis methods for communication research.

[B64-ejihpe-15-00179] Rahman M. M. (2023). Sample size determination for survey research and non-probability sampling techniques: A review and set of recommendations. Journal of Entrepreneurship, Business and Economics.

[B65-ejihpe-15-00179] Rahman M. M., Tabash M. I., Salamzadeh A., Abduli S., Rahaman M. S. (2022). Sampling techniques (probability) for quantitative social science researchers: A conceptual guidelines with examples. Seeu Review.

[B66-ejihpe-15-00179] Sandelowski M. (2000). Combining qualitative and quantitative sampling, data collection, and analysis techniques in mixed-method studies. Research in Nursing & Health.

[B67-ejihpe-15-00179] Sarstedt M., Henseler J., Ringle C. M. (2011). Multigroup analysis in partial least squares (PLS) path modeling: Alternative methods and empirical results. Measurement and research methods in international marketing.

[B68-ejihpe-15-00179] Sarstedt M., Ringle C. M., Hair J. F. (2021). Partial least squares structural equation modeling. Handbook of market research.

[B69-ejihpe-15-00179] Schmid A., Schoop M. (2022). Gamification of electronic negotiation training: Effects on motivation, behaviour and learning. Group Decision and Negotiation.

[B70-ejihpe-15-00179] Sheetal, Tyagi R., Singh G. (2023). Gamification and customer experience in online retail: A qualitative study focusing on ethical perspective. Asian Journal of Business Ethics.

[B71-ejihpe-15-00179] Shi Y., Haller A., Reeson A., Li X., Li C. (2025). Investigating the effects of nudges to promote knowledge-sharing behaviours on MOOC forums: A mixed method design. Behaviour & Information Technology.

[B72-ejihpe-15-00179] Smith K. A., Dennis M., Masthoff J., Tintarev N. (2019). A methodology for creating and validating psychological stories for conveying and measuring psychological traits. User Modeling and User-Adapted Interaction.

[B73-ejihpe-15-00179] Spector P. E. (2019). Do not cross me: Optimizing the use of cross-sectional designs. Journal of Business and Psychology.

[B74-ejihpe-15-00179] Srivastava P., Sehgal T., Jain R., Kaur P., Luukela-Tandon A. (2024). Knowledge management during emergency remote teaching: An interpretative phenomenological analysis of the transition experiences of faculty members. Journal of Knowledge Management.

[B75-ejihpe-15-00179] Stein C., Teubner T., Morana S. (2024). Designing a conversational agent for supporting data exploration in citizen science. Electronic Markets.

[B76-ejihpe-15-00179] Streukens S., Leroi-Werelds S. (2016). Bootstrapping and PLS-SEM: A step-by-step guide to get more out of your bootstrap results. European Management Journal.

[B77-ejihpe-15-00179] Suriagiri S., Norlaila, Wahyurudhanto A., Dalle J. (2022). Online vs. in-campus, comparative analysis of intrinsic motivation inventory, student engagement and satisfaction: A way forward for post COVID-19 era. Electronic Journal of E-Learning.

[B78-ejihpe-15-00179] Terkaj W., Urgo M., Kovács P., Tóth E., Mondellini M. (2024). A framework for virtual learning in industrial engineering education: Development of a reconfigurable virtual learning factory application. Virtual Reality.

[B79-ejihpe-15-00179] Thomas R. J., Masthoff J., Oren N. (2019). Can I influence you? Development of a scale to measure perceived persuasiveness and two studies showing the use of the scale. Frontiers in Artificial Intelligence.

[B80-ejihpe-15-00179] Van Zyl L. E., Ten Klooster P. M. (2022). Exploratory structural equation modeling: Practical guidelines and tutorial with a convenient online tool for Mplus. Frontiers in Psychiatry.

[B81-ejihpe-15-00179] Vinzi V. E., Chin W. W., Henseler J., Wang H. (2010). Handbook of partial least squares.

[B82-ejihpe-15-00179] Wachner J., Adriaanse M. A., De Ridder D. T. D. (2020). And how would that make you feel? How people expect nudges to influence their sense of autonomy. Frontiers in Psychology.

[B83-ejihpe-15-00179] Wagner R., Grimm M. S., Radomir L., Ciornea R., Wang H., Liu Y., Ringle C. M., Sarstedt M. (2023). Empirical validation of the 10-times rule for SEM. State of the art in partial least squares structural equation modeling (PLS-SEM).

[B84-ejihpe-15-00179] Wasko M. M., Faraj S. (2005). Why should I share? Examining social capital and knowledge contribution in electronic networks of practice. MIS Quarterly.

[B85-ejihpe-15-00179] Wei W., Zhang W., Wang C., Schrepp M. (2025). An empirical study of video duration on users’ discontinuous sage intention: Based on the TAM model. Design, user experience, and usability.

[B86-ejihpe-15-00179] Wenker K. (2022). A systematic literature review on persuasive technology at the workplace. Patterns.

[B87-ejihpe-15-00179] Wiafe I., Ekpezu A. O., Gyamera G. O., Winful F. B. P., Atsakpo E. D., Nutrokpor C., Gulliver S. R. (2024). Learning satisfaction in virtual reality: The role of persuasive design. International Journal of Human–Computer Interaction.

[B88-ejihpe-15-00179] Wong K. K.-K. (2013). Partial least squares structural equation modeling (PLS-SEM) techniques using SmartPLS. Marketing Bulletin.

[B89-ejihpe-15-00179] Yin J., Goh T.-T., Yang B., Xiaobin Y. (2021). Conversation technology with micro-learning: The impact of chatbot-based learning on students’ learning motivation and performance. Journal of Educational Computing Research.

[B90-ejihpe-15-00179] Yu Z., Xu W., Sukjairungwattana P. (2023). Motivation, learning strategies, and outcomes in mobile English language learning. The Asia-Pacific Education Researcher.

[B91-ejihpe-15-00179] Za S., Scornavacca E., Pallud J. (2022). Enhancing workplace competence acquisition through a persuasive system. Information Systems and E-Business Management.

[B92-ejihpe-15-00179] Zhang Q. (2023). Harnessing the power of technology: A systematic analysis of challenges, theoretical frameworks, and recommendations for K-12 online learning. SN Social Sciences.

[B93-ejihpe-15-00179] Zsifkovits M., Amplatz L., Triebner N., Utz J., Kornhuber J., Spitzer P. (2025). Randomized controlled trial of asynchronous vs. synchronous online teaching formats: Equal knowledge after training, greater acceptance and lower intrinsic motivation through asynchronous online learning. BMC Medical Education.

